# Nanomaterial-enabled drug delivery systems for circadian medicine: bridging direct rhythm modulation and chronotherapy

**DOI:** 10.1039/d5ra04137f

**Published:** 2025-09-05

**Authors:** Muhammad Zeeshan, Juncheng Hu, Chuan-Xi Mao, Almas Danish, Ying Xiong, Muhammad Sultan Irshad, Van-Duong Dao, Zhihua Liu

**Affiliations:** a State Key Laboratory of Biocatalysis and Enzyme Engineering, School of Life Sciences, Hubei University Wuhan P.R. China Ying.Xiong@hubu.edu.cn Zhihua_Liu@hubu.edu.cn; b Ministry of Education Key Laboratory for the Green Preparation and Application of Functional Materials, School of New Energy and Electrical Engineering, Hubei University Wuhan 430062 P.R. China muhammadsultanirshad@hubu.edu.cn; c Faculty of Biotechnology, Chemistry, and Environmental Engineering, Phenikaa School of Engineering, Phenikaa University Hanoi 12116 Vietnam duong.daovan@phenikaa-uni.edu.vn

## Abstract

Circadian rhythms are essential for maintaining health and homeostasis, and disruptions can lead to sleep disorders, metabolic diseases, cardiovascular diseases, and neurodegenerative conditions. Herein, we discuss the importance of circadian rhythms and the challenges in their regulation, highlighting the limitations of traditional drug delivery methods. Various nanomaterials such as liposomes, polymeric nanoparticles (PNPs), and mesoporous silica nanoparticles have unique physical and chemical properties. These characteristics allow them to deliver drugs to specific targets over a sustained period, which improves the effectiveness of treatments for circadian-related disorders. The article discusses how nanomaterials can be used to combine different treatment strategies to address the many challenges of disrupted circadian rhythms. By delivering several drugs at once, these systems can enhance treatment specificity and reduce side effects, offering a more effective approach to managing conditions such as sleep disorders and mood disorders associated with circadian misalignment. The potential for smart drug delivery systems (SDDSs) that respond to physiological cues, such as temperature or pH changes, is also explored, showcasing how these innovations could lead to more personalized and effective therapies.

## Abstract 2

Nanotechnology's dual potential in circadian medicine: smart drug delivery could help realign the body's clock or optimize therapy timing (chronotherapy), improving outcomes for major diseases.

## Graphical abstract

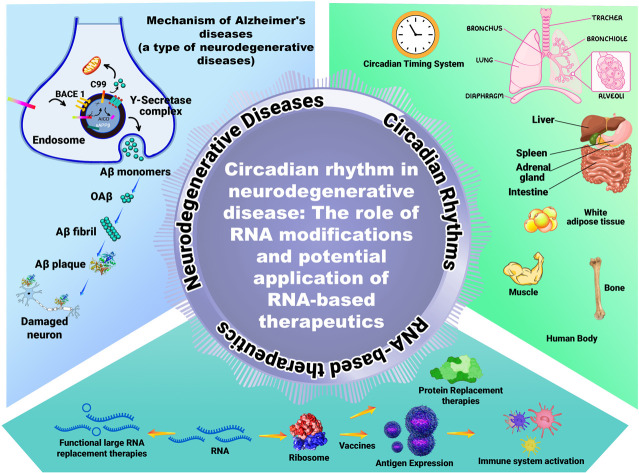

## 1. Introduction

Biological clocks, ticking silently within nearly every cell, orchestrate the ∼24-hour cycles known as circadian rhythms. Governed by a central pacemaker in the brain's suprachiasmatic nucleus (SCN) and synchronized primarily by light, these rhythms temporally organize a vast array of physiological processes, from sleep–wake cycles and hormone secretion to metabolism, immune function, and cellular proliferation.^1,2^ This intricate temporal coordination is essential for maintaining homeostasis and adapting to the predictable daily environmental changes. However, the modern world – characterized by artificial light, shift work, jet lag, and irregular eating patterns – frequently forces a misalignment between our internal clocks and the external environment, leading to circadian disruption.^3–7^ Furthermore, various disease states, including cancer, metabolic syndrome, neurodegenerative disorders, and psychiatric illnesses, are increasingly recognized to both cause and exacerbate by disrupted circadian rhythms.^8–12^

The growing understanding of the profound impact of circadian timing on health and disease has spurred the development of two major therapeutic avenues within the burgeoning field of circadian medicine. One approach, known as direct restoration or modulation of circadian rhythms, aims to directly correct underlying rhythm disturbances. This can be achieved through strategies like behavioral interventions, such as timed light exposure and sleep scheduling, as well as with medications known as chronobiotics (agents that can shift the body clock), such as melatonin or its agonists.^13^ Potentially, novel methods targeting core clock gene expression or signaling pathways could also be employed. The ultimate goal is to reset, stabilize, or strengthen weakened or misaligned internal rhythms. The second major therapeutic avenue, chronotherapy, focuses on aligning treatments with circadian rhythms.^14,15^ This strategy leverages the body's existing, often predictable, daily variations in physiology and pathophysiology.^16,17^ Chronotherapy involves administering conventional drugs like those for cancer, high blood pressure, or asthma at the time of day when they are most effective and cause the fewest side effects. The goal is to significantly improve treatment outcomes.^18^ To achieve this, it's essential to understand both the drug's pharmacokinetics (PK, how the body processes a drug) and pharmacodynamics (PD, how the body processes a drug) as well as the rhythmic nature of the disease process or target pathway.

While both approaches hold immense therapeutic promise, they face distinct but related hurdles. Direct modulation often struggles with targeted delivery of chronobiotics to relevant tissues (like the SCN or peripheral organs), achieving sustained release profiles that mimic natural rhythms, and overcoming biological barriers.^19^ Chronotherapy, particularly for systemic drug delivery, is hampered by the difficulty in achieving precise temporal control over drug release and concentration profiles *in vivo* using conventional formulations, often requiring complex dosing schedules with poor patient compliance.^19^

Here, we propose that nanotechnology provides a critical and convergent technological solution capable of overcoming key limitations inherent in both direct circadian modulation and chronotherapy. Nanomaterials, engineered structures with dimensions typically in the 1–100 nanometer range, possess unique physicochemical properties – including small size, large surface area, tunable surface chemistry, and novel optical or magnetic properties – that enable unprecedented interaction with biological systems.^20^ These properties can be exploited to design sophisticated platforms for diagnostics, imaging, and, crucially, therapeutic delivery.^21^ This review will connect these two therapeutic goals by highlighting how nanomaterials serve as a unifying enabling technology. We will explore how the adaptable nature of various nanomaterials allows for the design of systems tailored to these dual goals. The following sections will cover the molecular foundations relevant to both strategies, the principles of advanced drug delivery necessitated by circadian biology, the specific nanomaterials being investigated, and their potential applications in treating disorders linked to circadian disruption or amenable to chronotherapeutic optimization. The synergistic potential of using nanotechnology to either directly ‘fix’ the clock or perfectly ‘time’ treatments according to the clock underscores a new frontier in personalized and effective medicine.

**Fig. 1:**
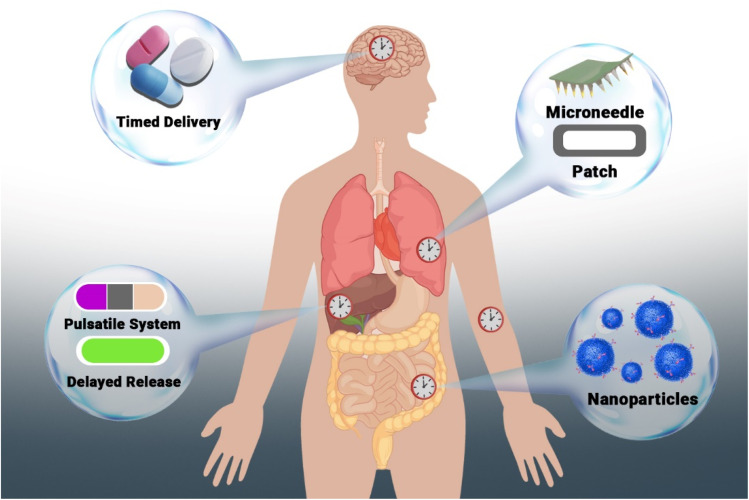
Schematic representation of tailored drug delivery strategies designed for targeted organ delivery and time-specific administration. The illustration depicts potential systems-such as timed release (brain), pulsatile or delayed systems (liver), microneedle patches (lungs), and nanoparticle-based delivery (intestine), as examples of emerging technologies that may, in the future, be aligned with circadian regulation of peripheral organs. This figure is conceptual and does not imply that organ-specific times for drug delivery systems are currently available or clinically established. It aims to highlight the growing potential of integrating circadian biology into advanced drug delivery design.

Although recent advances in chronotherapeutics have highlighted the importance of aligning drug delivery with organ-specific circadian rhythms, it is important to clarify that clinically validated, time-specific drug delivery systems for individual organs are still under development. Fig. 1 illustrates a conceptual framework of potential strategies-including times, pulsatile, delayed, nanoparticle-based, and microneedle drug delivery systems that could, in the future, be aligned with peripheral organs' clocks to optimize therapeutic efficacy. This schematic is intended to inspire future research into the design of circadian-informed drug delivery technologies and does not suggest that such systems currently exist for each organ.^22–25^

## 2. The molecular and cellular foundations of circadian rhythms and their role in health and disease

### 2.1 The molecular mechanisms and neural control of circadian rhythms

Circadian rhythms are governed by a complex interplay of genetic, molecular, and neural mechanisms. A set of core clock genes, such as *CLOCK*, *BMAL1*, *PERIOD* (*PER*), and *CRYPTOCHROME* (*CRY*), maintain rhythmic oscillations through an interlocking feedback loop.^26^ The SCN of the hypothalamus, the brain's master circadian pacemaker, receives direct input from the retina about light conditions.^27^ This input, mediated by intrinsically photosensitive retinal ganglion cells (ipRGCs) containing the photopigment melanopsin, helps synchronize the SCN with the external light–dark cycle, aligning internal rhythms with the environment. At the molecular level, circadian rhythms are generated through interconnected transcriptional–translational feedback loops (TTFLs). Briefly, the *CLOCK* and *BMAL1* proteins form a heterodimer that activates the transcription of *PER* and *CRY* genes. This activation leads to the production of *PER* and *CRY* proteins, which build up in the cell. Eventually, the *PER* and *CRY* proteins travel back into the cell's nucleus and turn off the *CLOCK*–*BMAL1* pair, shutting down their production. The proteins encoded by clock genes exhibit rhythmic fluctuations in their levels, accumulating during the day, peaking, and declining in a rhythmic pattern.^28^ This core TTFL is further stabilized and modulated by accessory loops involving nuclear receptors (*e.g.*, REV-ERBα/β, RORα/β/γ) and other regulatory factors, creating a robust yet plastic timekeeping mechanism.^21^ Each component of this intricate molecular machinery represents a potential molecular target for pharmacological interventions aimed at directly modulating clock function.

**Fig. 2:**
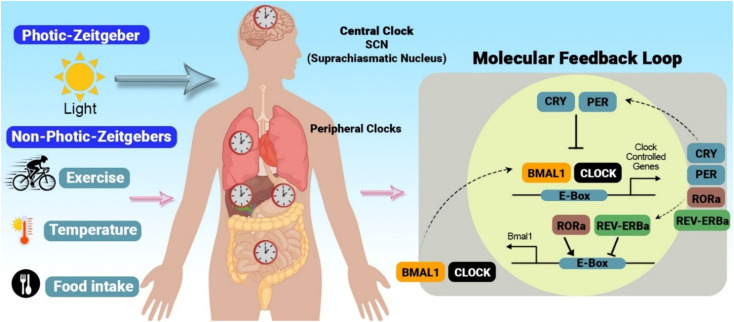
Integration of photic and non-photic zeitgebers by central and peripheral clocks. The suprachiasmatic nucleus (SCN), the body's central circadian pacemaker, coordinates peripheral clocks throughout the body *via* neural and humoral signals. Photic zeitgebers primarily influence the SCN, while non-photic zeitgebers, such as exercise, sleep, temperature, and food intake, can act on both the SCN and peripheral clocks. This integrated input ensures the alignment of internal physiological processes with the external environment. At the molecular level, the core circadian clock mechanism involves a transcription-translation feedback loop (TTFL). In this loop, the *BMAL1* and *CLOCK* proteins heterodimerize and bind to E-box elements in the promoters of target genes, including *PER* and *CRY,* as well as other clock-controlled genes (CCGs), thereby inducing their transcription. *PER* and *CRY* proteins then accumulate, heterodimerize, and translocate back into the nucleus, where they inhibit *BMAL1*–*CLOCK* activity, thereby suppressing their transcription. Concurrently, *BMAL1* and *CLOCK* also regulate the expression of the nuclear receptors RORα and REV-ERBα, which exert rhythmic activation and repression on *BMAL1* transcription, respectively, adding another layer of regulation to the core clock loop. The rhythmic expression of these core clock components and downstream CCGs drives circadian oscillations in a wide range of physiological processes, including metabolism, hormone secretion, and immune responses.

While the SCN serves as the master pacemaker, coordinating the body's overall circadian rhythmicity, nearly every tissue and organ has its own ‘peripheral’ clock that helps maintain local health and balance.^29,30^ These peripheral clocks, although capable of autonomous oscillations, are kept in sync (or ‘entrained’) by the SCN through neural and humoral signals, ensuring synchrony across the organism.^31^ Furthermore, they are responsive to local metabolic cues, integrating information from the environment and the internal milieu.^30^ For example, the liver clock is highly sensitive to feeding patterns, regulating glucose and lipid metabolism in a time-of-day-dependent manner.^32^ Similarly, the heart clock influences cardiac function and responsiveness to stress, with implications for cardiovascular health.^33^ Recent research has highlighted the importance of intercellular communication in coordinating rhythms across different tissues.^34^ Disruptions in the coupling between central and peripheral clocks or within peripheral clock networks have been linked to various disease states.^35^ Therefore, both the central SCN pacemaker and specific peripheral clocks represent key anatomical and tissue-specific targets for interventions seeking to restore a healthy, coordinated circadian rhythm. This multi-level network (Fig. 2) provides multiple strategic nodes for the development of therapies aimed at directly manipulating the circadian system.

### 2.2 Reciprocal regulation of the circadian clock and cell cycle

Beyond coordinating systemic physiology, the circadian clock controls cell division and genome maintenance, linking it to cancer development and treatment. This connection offers targets for clock modulation and the basis for chronotherapy, optimizing cancer treatment timing. Key cell cycle regulators at the G2-M (cell-division) and G1-S (DNA-synthesis) phases exhibit circadian expression, often regulated by the *CLOCK*–*BMAL1* heterodimer binding to DNA sequences called E-box elements.^36,37^ For instance, *CLOCK* directly regulates *Wee1*, a gene controlling mitotic entry, with its mRNA and kinase activity fluctuating daily, thus modulating cell division timing.^37^ Similarly, clock genes influence G1/S progression by regulating *Cdkn1a* (which encodes p21(WAF1/CIP1)), and disrupted clocks lead to abnormal p21 levels and proliferation issues.^36–39^ Furthermore, *CLOCK* regulates *c-Myc* and *Cyclin D1*, further solidifying the link between the circadian clock and cell cycle control. In turn, too much *c-Myc* can block *CLOCK*–*BMAL1* from activating *PER1*, suggesting a model where the biological clock drives c-Myc oscillations, while high c-Myc levels interfere with clock gene expression.^38^

The circadian clock also regulates DNA Damage Response (DDR), the system cells use to detect and repair DNA damage. This leads to daily fluctuations in how sensitive cells are to DNA-damaging stress (genotoxic stress), a fundamental principle in chronotherapy. DNA damage activates cell cycle checkpoints and genome surveillance. The *CLOCK*–*BMAL1* heterodimer transcriptionally regulates genes involved in the DDR.^40^ Furthermore, core clock components directly interact with key checkpoint pathways.^41^ For instance, *PER1* forms complexes with DNA-damage sensors ATM and CHK2, directly influencing their activity in response to DNA damage.^42^*CRY1*, interacting with TIMELESS in a circadian manner, modulates ATR-mediated DNA damage checkpoints, leading to rhythmic ATR activity.^43^ Notably, under genotoxic stress, stabilized *CRY1* promotes a specific type of DNA repair (homologous recombination), a process that has been linked to the progression of treatment-resistant prostate cancer.^43,44^ Disruptions in this interplay between circadian rhythms and the DDR can compromise genomic stability and impact the efficacy of chemo- or radiotherapy, underscoring the importance of chronotherapeutic strategies.^45^

A reciprocal regulatory loop between the tumor suppressor p53 and the core circadian protein *PER2* establishes another critical link between the circadian clock and the DDR. The p53 can repress the *PER2* gene transcription by competing with the *CLOCK*–*BMAL1* complex at the *PER2* promoter, a repression amplified under cellular stress.^46^ Conversely, *PER2* stabilizes p53 by forming a trimeric complex with p53 and another protein, MDM2, which normally flags p53 for destruction. By inhibiting MDM2, *PER2* allows p53 to build up, which in turn activates genes involved in stopping the cell cycle and repairing DNA.^47^ Additionally, *BMAL1* has been shown to bind to the p53 promoter, potentially activating its expression.^48^ This bidirectional regulation highlights the intricate integration of circadian rhythms with the cellular mechanisms that maintain genome stability.

**Fig. 3:**
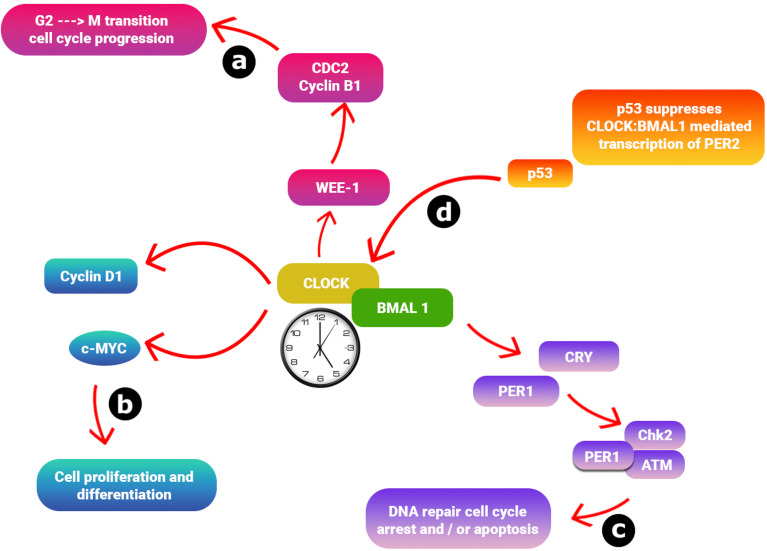
The core circadian clock interacts closely with components of the cell cycle. (a) The *CLOCK* complex drives the transcription of genes containing E-box elements in their regulatory regions, which include genes tied to both circadian rhythms and cell-cycle regulation. This complex directly regulates the cell-cycle-related gene Wee-1, which has three B-boxes in its promoter region. Wee-1 encodes a kinase that inactivates the CDC2/Cyclin B1 complex, thus playing a crucial role in managing the G2-M phase transition and overall cell-cycle progression. (b) *CLOCK* promotes the expression of Cyclin D1 and C-MYC, influencing cell growth and differentiation. (c) *PER1* can form a complex with ATM kinase and the checkpoint kinase Chk2, impacting DNA repair, cell-cycle arrest, and apoptosis. (d) Under both normal and stress conditions, p53 binds to the response element in the *PER2* promoter, overlapping with the *CLOCK* site, which suppresses the *CLOCK* transcription of *PER2*.

Collectively, these findings underscore the close connection between the circadian clock and the cell cycle, mediated by reciprocal molecular interactions between core clock proteins and key cell cycle regulators (Fig. 3). This temporal coordination may create rhythmic windows of cellular susceptibility or resistance during cell cycle progression, a concept particularly relevant to cancer pathogenesis, where uncontrolled cell division is a key feature.^49^ Supporting this, studies in mouse models have demonstrated that disruption of the p53-cell cycle-clock gene feedback loop is involved in the development of tumors.^50^ For example, a specific mutation in *p53* that is also linked to a human sleep disorder makes cells resistant to programmed cell death (apoptosis) and promotes cancer development in mice.^51^ Furthermore, in mouse cells with a mutated *PER2* gene, the timing of cell cycle genes was thrown off, suggesting that *PER2* is critical for keeping the cell cycle synchronized and that disruptions could lead to uncontrolled cell growth.^52^

### 2.3 Circadian disruption and sirtuins function in neurodegenerative and cardiovascular disease

**Fig. 4:**
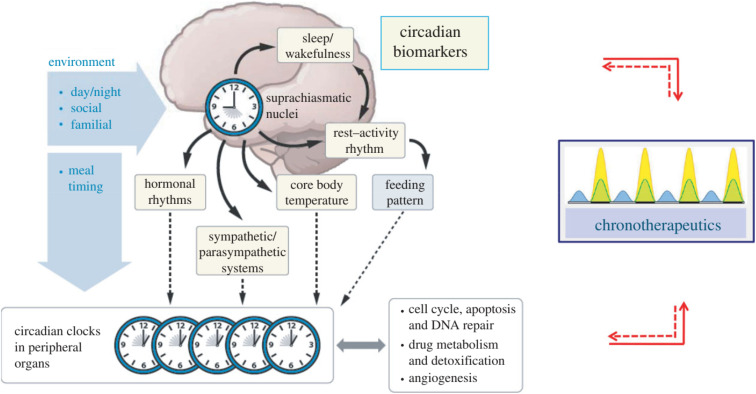
Pathological conditions associated with disruptions in the circadian rhythm. The diagram shows examples of how circadian rhythm disruption adversely influences brain function (cognition), heart physiology, reproduction, metabolism, hormone secretion, cancer, diabetes, and sleep. The arrows indicate up- and down-regulation. NAFLD non-alcoholic fatty liver disease, NASH non-alcoholic steatohepatitis, IR ischemia/reperfusion injury, HPA hypothalamic–pituitary–adrenal, HPG hypothalamic–pituitary–gonadal, HPT hypothalamic–pituitary–thyroid, LH luteinizing hormone, FSH follicle-stimulating hormone.^69^

Compelling evidence implicates circadian rhythm disruption as a significant contributor to the pathophysiology of neurodegenerative diseases, including Alzheimer's disease (AD) and Parkinson's disease (PD), as illustrated in Fig. 4. Age-associated deterioration in the integrity and output of the central circadian pacemaker, the SCN, often precedes or accompanies neurodegeneration. This leads to broken sleep patterns and weaker, shifted melatonin release-both of which are recognized hallmarks and potential risk factors for dementia.^53,54^ Genetic studies further strengthen this link, identifying associations between single-nucleotide polymorphisms (SNPs) in core clock genes (*e.g.*, *CLOCK*, *BMAL1*, *PER1*) and susceptibility to AD and PD.^55^ This genetic predisposition is further compounded by the dysregulation of melatonin secretion. Individuals afflicted with AD or PD exhibit a significant reduction in the amplitude of their melatonin rhythm, coupled with alterations in the timing of its release.^56^ These changes manifest clinically as sleep–wake disturbances, including excessive daytime sleepiness and delayed sleep onset.^57^ Interestingly, the temporal relationship between sleep disturbances and disease onset differs between PD and AD. In PD, sleep–wake disturbances often precede the emergence of motor or cognitive symptoms, making them potential diagnostic biomarkers. In contrast, sleep disruptions in AD generally become prominent after a formal diagnosis has been established.^58^ The neuroanatomical basis for these circadian disruptions is becoming clearer. Autopsy studies have revealed a substantial neuronal loss in the SCN in the brains of individuals with AD, which correlates with a reduction in the amplitude of motor activity rhythms observed before death.^59^ Furthermore, while clock gene expression persists in multiple brain regions of individuals with AD, the crucial synchronization of these rhythmic oscillators, both within and between regions, is profoundly disrupted.^60^ This loss of coordination may contribute to the cognitive and behavioral symptoms observed in AD.

Emerging evidence highlights a family of proteins called sirtuins (*SIRTs*), particularly the NAD+-dependent protein *SIRT1*, as crucial molecular players mediating the interplay between aging, metabolism, and circadian clock function. This connection helps explain how a decline in circadian function contributes to disease.^61^*SIRT1* levels and activity naturally decline with age, particularly within the SCN.^62^ This age-related decline mirrors the dampening of circadian rhythms observed in older individuals. Studies in rodents confirm this link: brain-specific *SIRT1* knockout recapitulates age-related circadian behavioral deficits, while *SIRT1* overexpression protects against them.^63^ Mechanistically, *SIRT1* directly interacts with and modifies core clock proteins like *CLOCK*, *BMAL1*, and *PER2*. By doing so, it helps control the timing, strength, and overall reliability of the daily clock rhythm.^64^ Its activity is tightly coupled to cellular NAD+ levels, which also decline with age, further contributing to the dampening of circadian rhythms.^65^ In the context of neurodegeneration, *SIRT1* dysfunction contributes significantly. Beyond its role in the SCN, *SIRT1* influences neurotransmitter systems implicated in disease, such as the dopaminergic system crucial for PD. *SIRT1* interacts with *CLOCK* at the promoter of the tyrosine hydroxylase (TH) gene, regulating its rhythmic expression and consequently dopamine synthesis.^66^ The age-related decline in *SIRT1* may therefore disrupt dopamine homeostasis. Furthermore, aging is associated with increased cytosolic iron, which can promote the oxidation of dopamine to the neurotoxic species dopamine quinone (DAQ).^67^ DAQ has been detected in PD-relevant cellular models.^68^ While the direct link needs further elucidation, it is possible that poor dopamine regulation caused by declining *SIRT1* could increase DAQ formation, contributing to the neurodegenerative cascade in PD.^66^

The circadian timing system (CTS) centers around the SCN, a hypothalamic master clock that orchestrates biological rhythms. This central pacemaker generates behavioral cycles and coordinates molecular clocks present in peripheral tissues through neural pathways, hormonal signals, and physiological outputs.^69^ A network of interconnected rhythmic indicators provides a window into the clock's function, including daily swings in cortisol and catecholamine levels, nightly melatonin secretion, patterns in the autonomic nervous system (*e.g.*, heart rate variability), body temperature cycles, and sleep–wake cycles.^70^ These synchronized rhythms not only maintain temporal organization across organs but also provide measurable biological markers of CTS function.^71^ Chronotherapeutic strategies leverage these daily variations to optimize treatment timing, particularly for anticancer therapies, by aligning medication administration with physiological rhythms to enhance drug effectiveness while reducing adverse effects. Importantly, treatments themselves can interact with the circadian system, creating complex feedback loops that must be considered when designing therapies.^72^

*SIRT1*'s influence extends prominently to the cardiovascular system, where it integrates circadian regulation with direct cardioprotective functions. Its ability to adjust the core clock machinery^73–75^ is vital, as chronic circadian disruption is a known stressor exacerbating cardiac dysfunction and metabolic imbalances.^68^ Beyond clock timing, *SIRT1* exerts broad cardioprotective effects on the heart: it enhances antioxidant defenses, modulates cardiac ion channels, inhibits atherosclerosis progression (*via* lipid metabolism and inflammation regulation), and promotes cardiomyocyte survival under stress by inhibiting apoptosis through interactions with p53 and FOXO proteins.^68,76^*SIRT1*'s activity is particularly important in mitigating damage from ischemia-reperfusion (I/R) injuries, which occur when blood flow is cut off from a tissue and then restored.^68^ Given that both *SIRT1* activity and NAD+ levels decline with age, diminishing these protective mechanisms, the NAD+/*SIRT1* pathway represents a critical target for age-related cardiovascular vulnerability.^77,78^

**Fig. 5:**
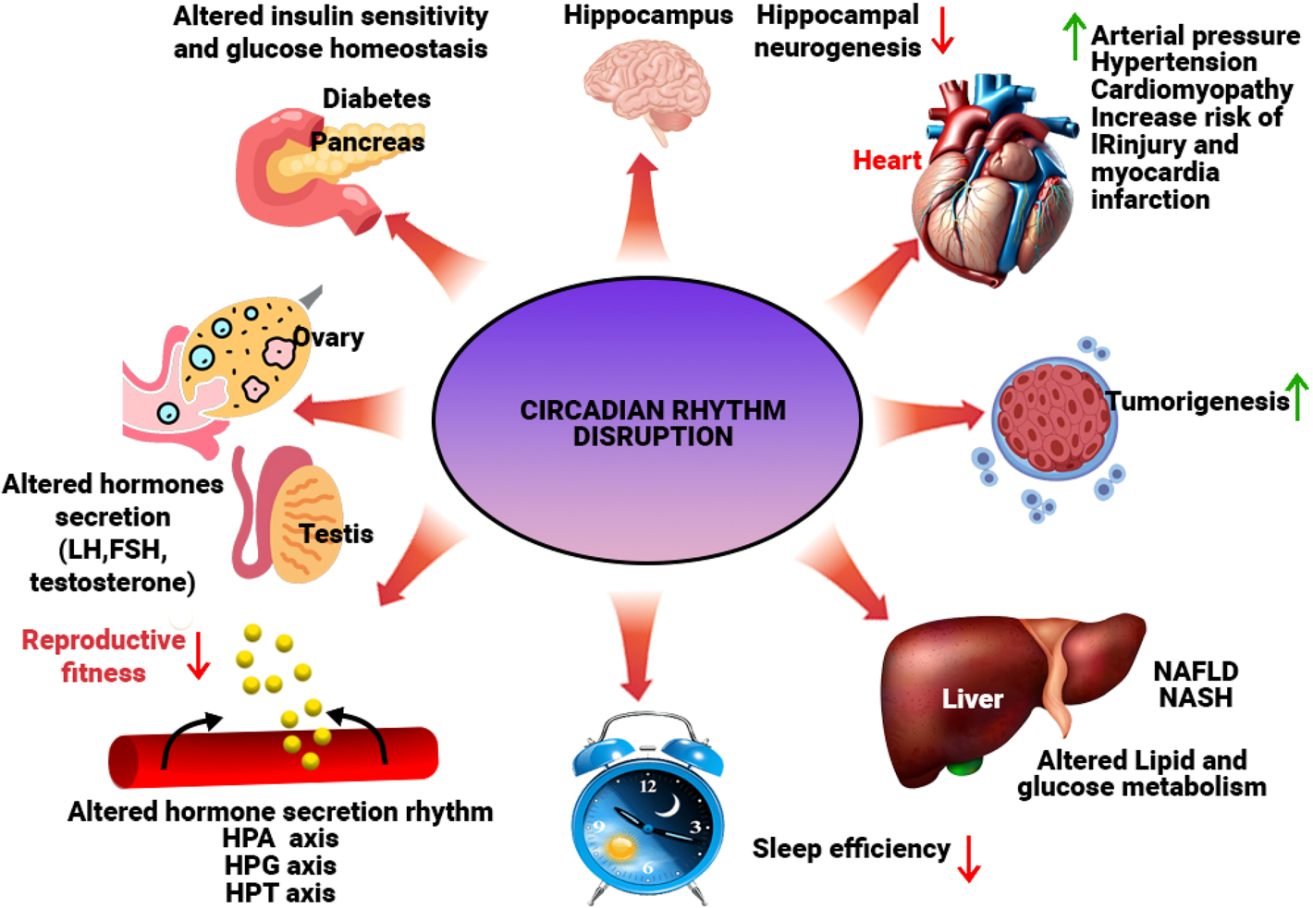
CTS: the CTS coordinates daily biological rhythms *via* SCN, a hypothalamic central pacemaker that synchronizes peripheral cellular clocks. These molecular clocks regulate detoxification, cell division, DNA repair, and other processes across 24-hour cycles, aligned with light–dark cues. Physiological rhythms (*e.g.*, hormone levels) serve as CTS biomarkers. Chronotherapy optimizes drug timing to match circadian peaks in tissue tolerance or drug efficacy, balancing treatment benefits with potential circadian disruptions.

Collectively, the intricate connections between circadian rhythms, *SIRT1* function, aging, and pathology in both the nervous and cardiovascular systems highlight *SIRT1* modulation as a promising therapeutic strategy. Restoring NAD+ levels with precursors like nicotinamide riboside (NR), which can rescue dampened circadian rhythms and improve cardiac function in preclinical models,^68^ could strengthen the circadian system and slow disease progression.^79^ Such approaches may simultaneously address neurodegeneration-relevant pathways involved in neurodegeneration (*e.g.*, dopamine homeostasis) and cardioprotective mechanisms (*e.g.*, inflammation, apoptosis). However, the effects of *SIRT1* can change depending on the biological context, so caution is needed, as excessive activation could be detrimental.^68^ Future research must prioritize elucidating the precise molecular links and conducting clinical trials to optimize strategies for targeting the *SIRT1*/NAD+ pathway, aiming to safely treat or prevent age-related neurodegenerative and cardiovascular disorders associated with circadian disruption (Fig. 5).

In summary, understanding how circadian rhythms work at the molecular and cellular level gives us critical insight into how they control metabolic balance, how diseases develop, and how the body responds to drugs. These basic mechanisms explain not only how a disrupted clock contributes to neurodegenerative, cardiovascular, and metabolic disorders but also how it affects the way drugs are absorbed, distributed, metabolized, and removed from the body. To translate this knowledge into better patient outcomes, there is a growing need for advanced drug delivery systems that can sync up with the body's natural rhythms, a core principle of chronotherapy. By aligning treatments with these internal clocks, such strategies promise to improve clinical results while reducing toxicity, marking a key shift from conventional medicine to time-guided medicine.

## 3. The need for advanced drug delivery for circadian rhythm regulation

### 3.1 The foundations of chronopharmacology and chronotherapy

Building upon the fundamental understanding of circadian rhythms and their pervasive influence on physiology, chronopharmacology emerges as a critical field bridging basic circadian biology and clinical therapeutics. Chronopharmacology investigates the intersection of circadian biology and pharmacology, examining how endogenous biological rhythms influence the PK and PD of medications.^80^ This field recognizes that key physiological processes governing drug absorption, distribution, metabolism, excretion, and target receptor sensitivity exhibit significant circadian oscillations.^81^ Consequently, the timing of drug administration can critically affect therapeutic efficacy and toxicity. Building directly on chrono-pharmacological principles, chronotherapy involves the strategic timing of drug administration to align with specific phases of the circadian cycle. The objective is to optimize therapeutic outcomes by administering medications when target pathways are most responsive, disease activity peaks, or drug tolerance is highest.^82,83^ This approach aims to maximize desired effects while minimizing adverse events, representing a crucial refinement beyond selecting what drug to use, focusing instead on when it should be administered for optimal impact.

The rationale for chronotherapy is strengthened by the wide-ranging impact of circadian rhythms on health and disease, including metabolic disorders like obesity and diabetes,^84^ cardiovascular diseases,^85^ and cancer.^86,87^ For instance, aligning therapeutic interventions with endogenous metabolic rhythms is a recognized strategy for managing metabolic disorders,^88^ which shows how timed drug delivery can improve treatment effectiveness in many different diseases.^83^

This growing understanding drives the need for advanced drug delivery systems that can put chronotherapy into practice. Chronotherapeutic drug delivery systems (CDDS) are engineered to release therapeutic agents in a programmed schedule, matching their availability to the best time windows based on the body's daily rhythms.^89,90^ Such precise temporal control is essential for realizing the full potential of chronotherapy. For example, in oncology, aligning chemotherapy delivery with circadian rhythms, potentially guided by biomarkers like skin surface temperature oscillations, aims to improve the therapeutic index by enhancing both tolerability and efficacy.^87^ Key benefits associated with successful chronotherapy implementation often include improved treatment response, reduced required dosages, and minimized side effects, particularly in chronic disease management.^91,92^ Furthermore, by enabling the monitoring of individual circadian patterns and tailoring administration schedules accordingly, chronotherapy represents a significant advancement towards personalized medicine.^93^

### 3.2 Smart drug delivery systems (SDDSs) enable temporal and targeted control

While chronopharmacology establishes the why of time-controlled drug delivery, SDDSs provide the how.^94^ Traditional drug delivery methods, which often aim for a steady, constant drug level, are not ideal because they ignore the dynamic, daily changes in the body that are controlled by circadian rhythms. SDDSs are a key technological advance because they provide the precise timing needed for chronotherapy.^95^ These systems are engineered to sync drug delivery with the body's natural daily rhythms, which can improve treatment effectiveness and reduce side effects.^95,96^

A defining characteristic of many SDDSs is their ability to sense and respond to specific environmental cues, triggering drug release precisely when needed.^97^ One major class of SDDSs is stimulus-responsive delivery systems (SRDSs). SRDSs incorporate materials that undergo physical or chemical changes in response to specific stimuli, leading to drug release. These triggers fall into two main types: internal and external. Internal, or physiological, triggers originate from within the body and are often associated with disease states or normal physiological variations. Examples include changes in pH, higher levels of certain enzymes, shifts in the cell's chemical balance (redox potential), or the presence of specific biological molecules.^98^ External triggers, on the other hand, are applied from outside the body, offering greater control over the timing and location of drug release. Examples include light, temperature, ultrasound, and magnetic fields.^98,99^

To align SDDSs with circadian rhythms, two main strategies stand out. The first involves tapping into the body's natural cycles. Many physiological parameters exhibit robust circadian oscillations. These include body temperature, hormone levels, and the activity of certain enzymes. SRDSs can be designed to respond to these endogenous rhythmic changes, releasing their drug payload at specific phases of the circadian cycle. For instance, a system could release a blood pressure medication in response to the typical morning surge, or it could release a cancer drug when tumor cells are dividing most rapidly a process timed by the circadian clock.^100^ The second approach uses external triggers to impose a rhythm. External triggers offer a high degree of control over drug release timing. By applying these triggers in a carefully timed manner, it's possible to mimic or reinforce natural circadian rhythms, or even to create entirely new therapeutic rhythms. For example, a light-responsive drug delivery system could be activated at a specific time each day, ensuring consistent and time-optimized drug delivery.^101^

### 3.3 Overcoming bioavailability barriers *via* transdermal and non-oral delivery routes

**Fig. 6:**
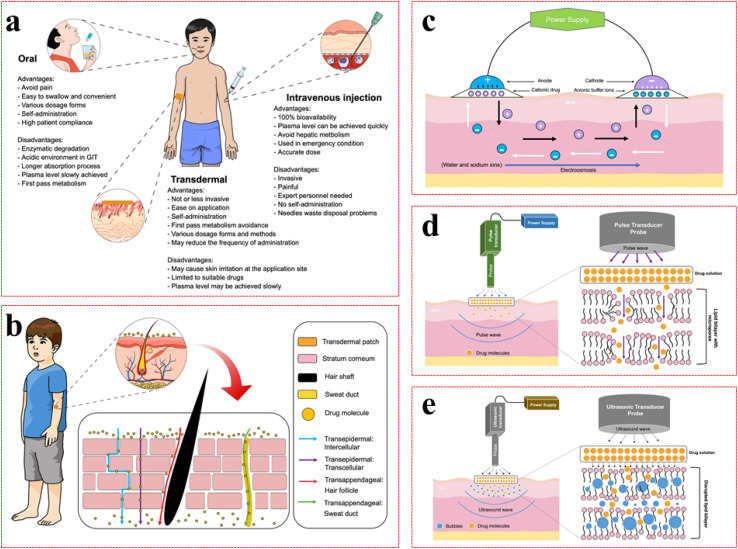
Overview of drug administration routes and physical enhancement methods for transdermal delivery. (a) Comparison of oral, intravenous (IV), and transdermal administration, noting their key trade-offs regarding convenience, bioavailability, invasiveness, and metabolism. (b) Primary skin permeation pathways: the transepidermal (intercellular and transcellular) and transappendageal (*via* hair follicles and sweat ducts) routes. (c) The mechanism of iontophoresis, where an electric current drives charged drug molecules across the skin. Black arrows indicate cationic drug movement from solution to the cathode, while white arrows represent anion movement from buffer solution to the anode. (d) Schematic diagram illustrating the use of electroporation to enhance transdermal drug delivery. Electrical pulses temporarily increase skin permeability, facilitating drug penetration into the dermis layer. (e) Schematic illustration of sonophoresis-assisted transdermal drug delivery, where ultrasound enhances skin permeability, facilitating drug delivery into the dermis.^102^

While SDDS provides mechanisms for temporal control, the route of administration significantly influences the feasibility and effectiveness of chronotherapy. Non-oral routes, particularly transdermal delivery, offer distinct advantages over conventional oral administration for implementing controlled-release strategies. A primary benefit is bypassing the digestive system and the liver, where drugs often get broken down before they can reach the bloodstream (a process known as first-pass metabolism). Avoiding this breakdown can greatly improve how much of the drug gets into the body (its bioavailability), leading to more predictable drug levels. This may allow for lower doses and less frequent administration both of which help patients stick to their treatment plan, especially for chronic diseases that require circadian timing (Fig. 6).^102^

Transdermal delivery, where a drug passes through the skin, is a great match for SDDSs designed for slow and controlled release.^101^ However, the skin's principal barrier, the stratum corneum, impedes the passage of many molecules. Overcoming this requires advanced formulation and delivery technologies.^101^ Early “first-generation” techniques to improve skin penetration included changing the drug's properties (like its solubility), using tiny carriers (like liposomes), adding chemicals that temporarily loosen the skin's barrier, or using physical methods like a weak electrical current (iontophoresis) or ultrasound (sonophoresis) to actively push the drug through.^103,104^ More recent advancements leverage nanomaterials and sophisticated micro-fabrication techniques. Nanomaterials, due to their unique physicochemical properties such as high surface area-to-volume ratios and tunable surface characteristics, can improve drug loading, enhance penetration into or through the skin barrier, and facilitate controlled release kinetics. For example, incorporating permeation enhancers like d-α-tocopherol polyethylene glycol 1000 succinate (TPGS) and alpha-lipoic acid (ALA) into a transdermal film significantly increased vinpocetine penetration.^105^ Similarly, nanocarrier systems like methoxy-poly(ethylene glycol)-di-hexyl-substituted lactide (MPEG-dihex-polylactic acid (PLA)) micelles have demonstrated enhanced accumulation of antifungal drugs in the skin.^105,106^

Among the most promising advancements in transdermal delivery is microneedle technology. Microneedles are arrays of micron-scale needles that painlessly create temporary microchannels in the skin, effectively bypassing the stratum corneum barrier.^107^ This allows for the delivery of a wide range of molecules, including both low-molecular-weight drugs and larger biotherapeutics (proteins, nucleic acids) that would otherwise be unable to penetrate the skin.^108^ Microneedle platforms also offer opportunities for intradermal drug delivery and even for therapeutic drug monitoring, making them highly versatile. By combining the benefits of non-oral routes with the precise timing of SDDSs and new technologies like microneedles, advanced delivery systems give us the tools needed to unlock the full potential of chronotherapy.

We have established that the timing of drug delivery is just as important as its composition. Smart delivery systems provide the temporal control needed for chronotherapy, but even the most sophisticated timing is useless if the drug cannot get past the body's natural defenses to reach its target. This is where nanotechnology offers a transformative solution, providing a powerful toolkit to solve both the timing and delivery challenges at once. By engineering materials at the nanoscale, scientists can create platforms that not only release drugs on a precise schedule but also navigate the body to specific cells or tissues. In the next section, we will dive into the specific nanomaterials from lipid-based carriers to PNPs that are making this new era of time-guided, targeted medicine a reality.

## 4. Nanomaterials as enabling platforms for circadian rhythm regulation and chronotherapy

### 4.1 Advantages of nanotechnology-based drug-delivery systems in circadian rhythm modulation

Nanotechnology has revolutionized drug delivery by providing unprecedented control over drug release kinetics, biodistribution, and targeting. Nanoparticle-based drug delivery systems (NDDSs), which are typically smaller than 100 nanometers, use a variety of biodegradable materials, including polymers, lipids, and metals, to carry or attach to drugs.^109^ This nanoscale engineering enables both targeted drug delivery and controlled release, significantly improving upon traditional drug formulations.

**Fig. 7:**
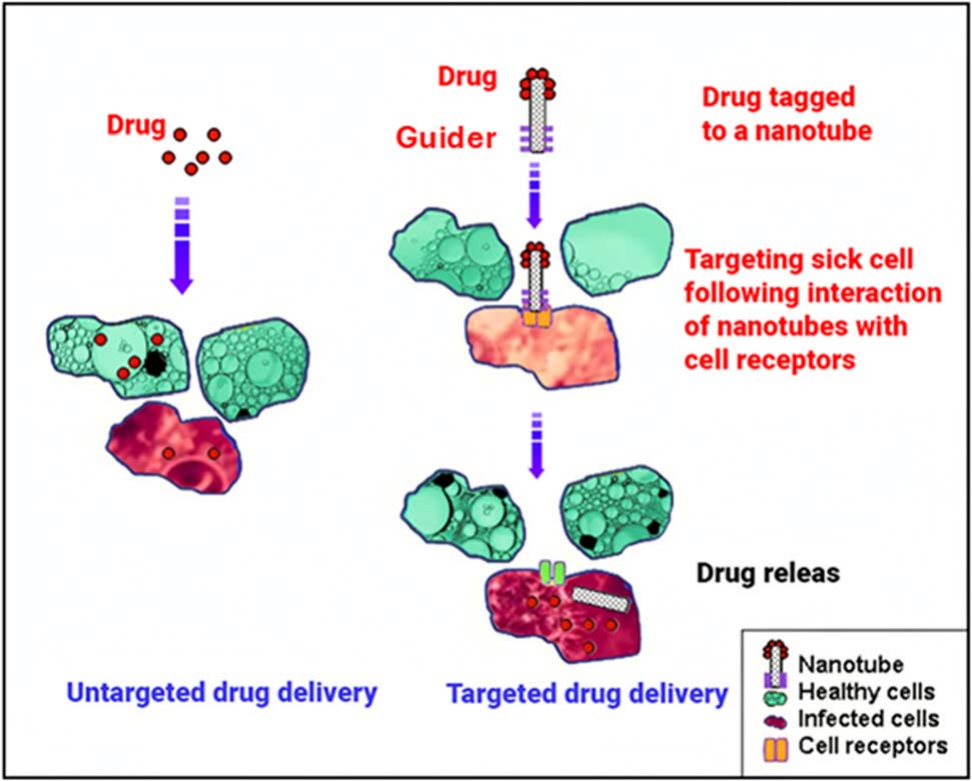
Schematic representation of targeted and untargeted drug delivery systems. The targeted system directs therapeutic agents specifically to diseased cells using ligands or stimuli-responsive carriers, enhancing efficacy and reducing side effects. In contrast, the untargeted system distributes drugs non-specifically throughout the body, potentially affecting both healthy and diseased cells.

A primary advantage of NDDSs is their ability to achieve site-specific targeting, minimizing off-target effects and reducing systemic toxicity (Fig. 7). This targeting can happen in two ways. The first is passive, where nanoparticles take advantage of leaky blood vessels commonly found in tumors to accumulate there a phenomenon known as the enhanced permeability and retention (EPR) effect.^110^ The second is active, where the nanoparticle surface is coated with molecules (like antibodies or peptides) that act like keys, binding only to specific locks (receptors) that are more common on target cells.^111^ Various NDDS platforms, including porous silicon nanoparticles,^112^ liposomes, and self-assembled micelles,^113^ have demonstrated improved drug accumulation in target tissues, such as tumors, *via* these mechanisms (Table 1).

**Table 1:** Various nanomaterials are used for cancer treatment

Nanomaterials for cancer treatment	Advantages	Reported literature examples with references
Magnetic nanoparticle	(i) Resonance of magnetism imaging characteristics for use in theranostics	(i) Iron oxide nanoparticle carrying MHC-Ig dimer and anti-CD28 (ref. 246)
	(ii) Targeted therapy	
	(iii) Hyperthermia applications	
Liposome	(i) The most well-defined nanotechnology in the medical field	(i) Liposomal antigenic protein^247^
	(ii) Tumor immunotherapy's most researched medication delivery method	(ii) Liposomal doxorubicin (Doxil)^248^
	(iii) There are industrial guidelines for liposome drug products from the U.S. FDA	(iii) PEGylated liposome-anchored combinatorial immunotherapy^249^
Biomimetic nanoparticles	(i) Potentially low toxicity	(i) Viral protein^250^
	(ii) Longer circulation time	(ii) Biomimetic protein^251^
	(iii) Targeted delivery	(iii) Cancer cell membrane-coated NPs^252^
Polymeric micelles	(i) High drug loading capacity and surface characteristics that are simple to adjust	(i) Linear polyethyleneimine-based (PEI-based) nano-micelles^253^
	(ii) Ideal for transporting chemicals that are poorly soluble in water	(ii) Thioguanine-loaded polymeric micelles^254^
		(iii) T-cell-targeting nanoparticles^255^
		(iv) Micelles composed of poly(ethylene oxide)-*block*-poly- (α-carboxylate-ε-caprolactone) and STAT3 inhibitor^256^
		(v) EGCG micellar nanocomplex^257–259^
Inorganic nanoparticles	(i) Large surface area, small size, and surface characteristics that are simple to modify	(i) Copper sulfide CuS NPs^260^
	(ii) High stability	(ii) Inorganic silica NPs^261^
		(iii) Gold NPs^262^
Nanoparticles with iron oxide core and zinc oxide shell	(i) Using imaging agents and genes of interest simultaneously	(i) Multipurpose core–shell nanoparticle for cancer immunotherapy based on dendritic cells^263^
	(ii) Photodynamic and photothermal aspects to kill cancer cells	
Cholesterol-bearing pullulan-based hydrogel	(i) Self-assembling, colloidally stable, and comparatively monodisperse	(i) Interleukin-12 nanogel^264^
	(ii) Sustained drug release	

Furthermore, NDDSs have proven particularly effective in overcoming multidrug resistance (MDR) in cancer, a major obstacle to successful chemotherapy. MDR is often mediated by P-glycoprotein, a transmembrane efflux pump that actively removes chemotherapeutic drugs from cancer cells.^114^ Nanoparticles can bypass this efflux mechanism, delivering their therapeutic payload directly into resistant cancer cells.^115^ This capability has been demonstrated with a wide range of anticancer drugs, including paclitaxel (*e.g.*, abraxane®, also approved and effective for pancreatic cancer),^116,117^ doxorubicin, 5-fluorouracil, and dexamethasone, successfully formulated using various nanomaterials.^118^ For example, nanoparticles made of poly(lactic-*co*-glycolic acid) (PLGA) provide sustained release of the drug dexamethasone and stop the growth of certain blood vessel cells.^119^ Dexamethasone works by binding to receptors inside the cell, which in turn changes how certain genes are expressed.^120^ Paclitaxel-loaded nanoparticles have shown efficacy in paclitaxel-resistant colorectal tumors, addressing both drug resistance and paclitaxel's poor solubility.^121^ The clinical success of albumin-bound paclitaxel (abraxane),^122^ an approved nano-suspension for breast cancer, exemplifies the transformative potential of nanomedicine. Abraxane eliminates the need for toxic solvents, reducing associated side effects,^123^ and clinical trials have demonstrated a doubled response rate compared to traditional paclitaxel formulations.^124^

**Fig. 8:**
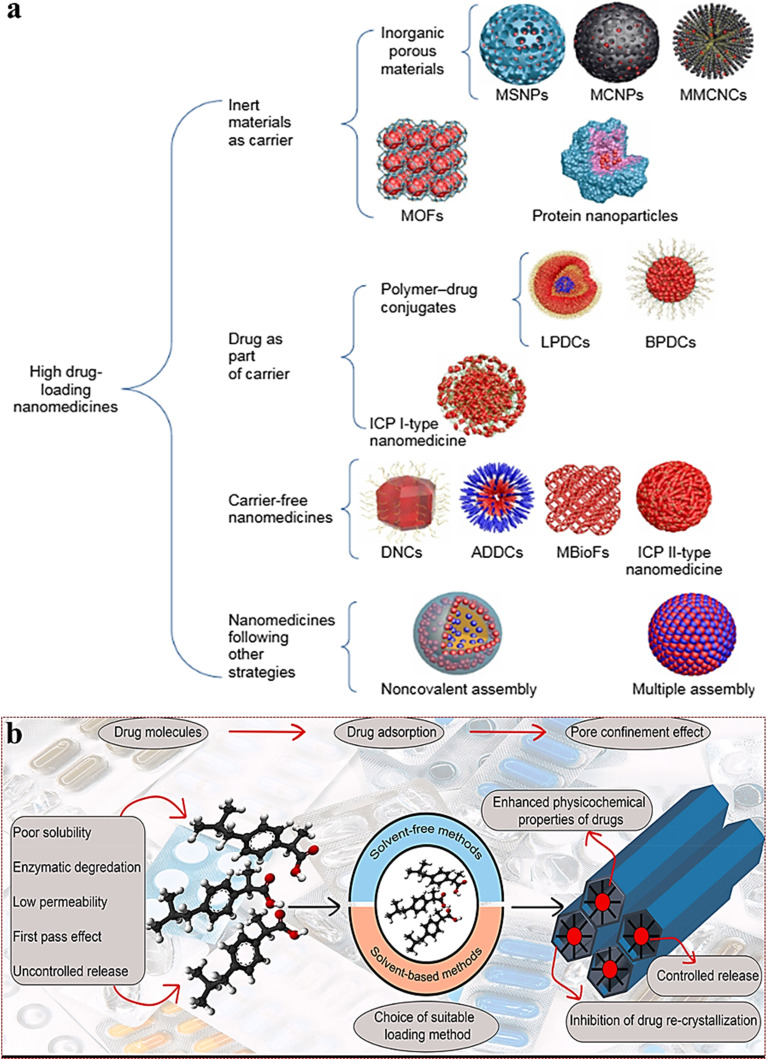
Strategies for high drug loading in nanomedicines and mesoporous carrier-based approaches. (a) Strategies for achieving high drug loading in nanomedicines include the use of inert carriers, drug-integrated systems, carrier-free formulations, and self-assembled nanostructures such as MSNPs (mesoporous silica nanoparticles), MCNPs (mesoporous carbon nanoparticles), MMCNCs (mesoporous magnetic colloidal nanocrystal clusters), MOFs (metal–organic frameworks), LPDCs (linear polymer–drug conjugates), BPDCs (branched PDCs), ICP (infinite coordination polymer), DNC (drug nanocrystals), ADDCs (amphiphilic drug–drug conjugates), MBioFs (metal–biomolecule frameworks).^125^ (b) A schematic illustration depicting drug loading into mesoporous carriers through solvent-free and solvent-based methods, enhancing solubility, improving stability, and enabling controlled release through pore confinement.^126^

A nanoparticle's drug loading capacity—how much drug it can carry is a critical factor in determining whether it can be successfully used in patients. Many conventional nanocarrier systems, including those based on PLGA and PLA, often exhibit relatively low drug loading efficiencies-typically less than 10 wt% of the total nanoparticle mass-posing a major limitation (Fig. 8a).^125^ This means that a large amount of carrier material must be given to deliver a small amount of drug, which increases the risk of side effects from the carrier itself. In contrast, inorganic nanocarriers such as mesoporous silica nanoparticles offer much higher drug-loading capacities, typically ranging between 40–70 wt%. For instance, ibuprofen-loaded mesoporous calcium silicate nanoparticles have achieved a drug content of ∼69 wt% with a loading efficiency of ∼86 wt%.^126^ Similarly, mesoporous silica nanoparticles have been employed for the encapsulation of anticancer drugs like cisplatin and caffeic acid, achieving drug contents around 44–48 wt%.^125^ Drug nanocrystals represent another class of delivery systems with the potential for up to 100 wt% drug loading, as they consist almost entirely of active pharmaceutical ingredients (Fig. 8b).^126^ Though typical values hover around 20 wt%, paclitaxel nanocrystals have demonstrated improved efficacy and reduced systemic toxicity *in vivo*.^125^ Low drug-loading formulations (*e.g.*, <10 wt%) often require large doses of nanoparticle carriers to achieve therapeutic drug concentrations. This elevates the excipient load, increases the risk of adverse reactions, and complicates regulatory and manufacturing pathways. Conversely, high drug-loading nanoparticles reduce the total mass of carrier required, improving the therapeutic index, reducing side effects, and potentially lowering costs.^127^ This effect is exemplified in studies using PRINT polymer nanoparticles loaded with docetaxel, where a lower loading (9 wt%) showed better plasma and tumor exposure, with lower off-target accumulation, compared to a 20 wt% formulation.^127^ Importantly, nanoparticle formulations do not usually reduce the total amount of drug a patient needs; instead, they reduce the amount of carrier material needed to deliver that drug. Thus, while the dose of the drug itself stays the same, high-loading designs make the delivery more efficient, improving overall safety, manufacturability, and cost-effectiveness.^128^ Overall, these advancements in nanomedicine design are critical for achieving clinically viable formulations with optimized therapeutic indices.

Crucially for chronotherapy, the precise control over drug release that NDDSs provide is a perfect match for the goals of circadian medicine.^129^ The ability to engineer nanoparticles for specific release profiles triggered, sustained, or pulsatile is fundamental to developing time-targeted delivery systems. Such systems can synchronize drug availability with endogenous biological rhythms, matching peak drug exposure to optimal therapeutic windows identified through chronopharmacology. For example, dendrimer-based nano systems have been designed to release paclitaxel according to circadian-defined schedules.^129^ Moreover, coordinating nanoparticle-mediated delivery with circadian timing has been shown to enhance drug targeting and cellular uptake (*e.g.*, transfection efficiency) in cancer models.^130^ This combination of nanotechnology's precise delivery with the insights of chronopharmacology creates a powerful synergy, paving the way for safer and more effective treatments.^82^

### 4.2 Lipid nanoparticles as versatile platforms for nucleic acid and small molecule delivery

Lipid nanoparticles (LNPs) have emerged as a leading platform in nanomedicine, particularly valued for their biocompatibility and efficiency in delivering nucleic acids (pDNA, siRNA, mRNA) and other bioactive molecules, including nutraceuticals relevant to metabolic and circadian health.^131^ Their effectiveness stems from their biocompatibility, their ability to protect sensitive cargo from degradation, and their capacity to facilitate efficient cellular uptake and, crucially, escape from cellular compartments called endosomes a key step needed to deliver nucleic acids inside a cell.

The careful design of LNPs involves fine-tuning their lipid ingredients and physical and chemical properties to get the best results. A typical LNP recipe includes an ionizable lipid (which is key for packaging nucleic acids and helping them escape the endosome), helper lipids (to help with cell entry), cholesterol (for structure), and PEGylated lipids (to provide stability and control how long the LNP circulates in the body).^132,133^ The choice of ionizable lipid is especially important because once inside the cell's acidic endosome, it becomes charged. This charge allows it to break open the endosome's wall, releasing the drug into the cell's main compartment (the cytoplasm).^134^ Beyond composition, parameters such as particle size, surface charge, and the potential inclusion of targeting ligands significantly influence LNP stability, biodistribution, cellular interactions, and ultimately, therapeutic performance.^135^

Further refinements in LNP technology aim to enhance functionality and safety. For instance, incorporating bioreducible cationic polymers can facilitate siRNA release specifically within the reducing environment of the cytoplasm, potentially mitigating cytotoxicity associated with non-biodegradable cationic materials.^136^ The inclusion of polyethylene glycol (PEG) *via* PEGylated lipids is a common strategy to help the LNP avoid detection by the immune system, keep it stable, and prolong the time it circulates in the bloodstream.^137^ Related lipid-based systems, such as solid lipid nanoparticles (SLNs) and nanostructured lipid carriers (NLCs), offer alternative platforms particularly suited for enhancing the stability and bioavailability of poorly soluble small-molecule drugs and certain biomolecules.^138^

**Fig. 9:**
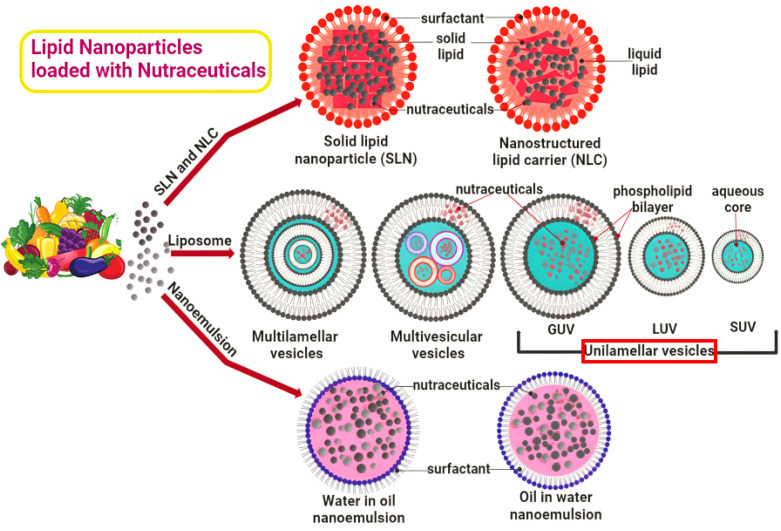
Architectures of lipid nanoparticle platforms for nutraceutical delivery. The diagram summarizes the key lipid-nanoparticle (LNP) platforms employed to package and deliver nutraceuticals. Each platform is defined by its core architecture and composition, which in turn control its affinity for hydrophilic *versus* lipophilic actives. Solid-core carriers include solid lipid nanoparticles (SLNs), built on a crystalline lipid matrix, and nanostructured lipid carriers (NLCs), which combine solid and liquid lipids to generate a less-ordered matrix that boosts loading efficiency and curbs drug leaching. Liposomes consist of phospholipid bilayers that form vesicles with an internal aqueous space; water-soluble nutraceuticals occupy this core, while oil-soluble compounds embed within the bilayer. Liposomal systems are further categorized by lamellarity—unilamellar, multilamellar, or multivesicular vesicles—and by diameter, from small (SUVs) to large (LUVs) and giant (GUVs). Nanoemulsions are kinetically stable colloidal dispersions that function as oil-in-water emulsions for lipophilic cargos or water-in-oil emulsions for hydrophilic cargos.

A key strength of LNPs is their capacity to encapsulate a wide array of payloads. Beyond delivering single nucleic acid species, they uniquely enable the co-delivery of multiple components, which is essential for complex applications like CRISPR/Cas9 gene editing, requiring simultaneous delivery of guide RNA and Cas9 mRNA or plasmid DNA.^139^ LNPs also effectively address the formulation challenges of many nutraceuticals (*e.g.*, polyphenols, vitamins) that suffer from poor solubility, stability, or absorption, thereby enhancing their bioavailability and potential therapeutic impact^131^ (Fig. 9). Their ability to accommodate both hydrophilic and hydrophobic molecules underscores their broad applicability.^131^

The intersection of LNP technology and circadian biology presents intriguing therapeutic possibilities. Leveraging their established efficiency in non-viral gene delivery,^133,140^ LNPs offer a powerful modality to directly influence the molecular clockwork. Core clock genes like *BMAL1* can potentially be targeted using LNP-delivered gene editing tools (*e.g.*, CRISPR-Cas9) or other gene-modulating nucleic acids (*e.g.*, siRNA) to correct circadian dysfunction.^141^ Furthermore, the delivery and efficacy of LNPs themselves may be subject to chronopharmacological principles. Circadian rhythms influence numerous cellular processes, including endocytosis and gene expression, suggesting that the timing of LNP administration could impact therapeutic outcomes.^142^ Optimizing LNP delivery schedules based on circadian timing may therefore enhance efficacy, particularly for diseases like cancer, where tumor biology exhibits significant circadian variation.^143,144^

### 4.3 Polymeric nanoparticles are tunable systems for sustained and targeted delivery

Polymeric nanoparticles (PNPs) are a highly adaptable and widely used platform in nanomedicine, offering major advantages for delivering drugs to treat circadian rhythm disorders.^145^ Their value comes from being biodegradable and safe for the body, having a large drug-carrying capacity, and their ability to improve the solubility and stability of the drugs they carry.^146,147^ Critically, the physical and chemical properties of PNPs can be precisely engineered to achieve controlled and sustained drug release profiles, making them exceptionally suitable for chronotherapeutic strategies that demand temporal precision in drug delivery.

This adaptability extends to the diverse range of therapeutic molecules PNPs can accommodate, including proteins, peptides, small molecules, and nucleic acids.^148^ Such versatility is advantageous for addressing complex conditions like circadian disorders, which may necessitate targeting multiple pathways or co-delivering synergistic agents. Beyond simply releasing drugs as the polymer carrier breaks down, PNPs can be designed as “smart” systems that respond to specific signals. These advanced platforms release their payload upon encountering specific environmental triggers such as changes in pH, temperature, enzyme levels, or redox potential allowing drug delivery to be synchronized with endogenous physiological cycles or pathological states.^77,79,149^

However, using the body's natural temperature rhythm as a trigger is challenging. While body temperature fluctuates by 1–2 °C over a 24-hour cycle, this small change is usually not enough to activate most temperature-sensitive (thermoresponsive) drug delivery systems.^150,151^ Thermoresponsive polymers like poly(*N*-isopropylacrylamide) (PNIPAM) work by having a “switching point” temperature called the lower critical solution temperature (LCST). Above this temperature, the polymer changes from being hydrophilic to hydrophobic, which pushes the drug out. The LCST can be adjusted, but most systems require temperatures well above the normal body temperature of ∼37 °C to release drugs effectively. For instance, one study found that cisplatin-loaded hydrogels released only about 22% of their drug at 37 °C but released 32% at 42 °C, showing that a significant temperature jump is needed.^150,151^ Therefore, the body's normal 1–2 °C daily temperature swing is not enough to flip the switch on most conventional thermoresponsive nanoparticles. Overcoming this may require designing highly sensitive thermal switches, a research area that remains largely unexplored. Alternatively, chronoparmacological approaches offer practical strategies. For example, For example, when mice were given nanoparticles loaded with the painkiller dalargin, the drug was much more effective when administered at 8:00 AM (90% effect) compared to 8:00 PM (70% effect), showing that timing alone has a major impact on efficacy.^152^ While synchronizing drug release with physiological temperature rhythms remains a challenge, aligning drug delivery with the body's other daily rhythms is a proven way to improve treatment results.^153^

**Fig. 10:**
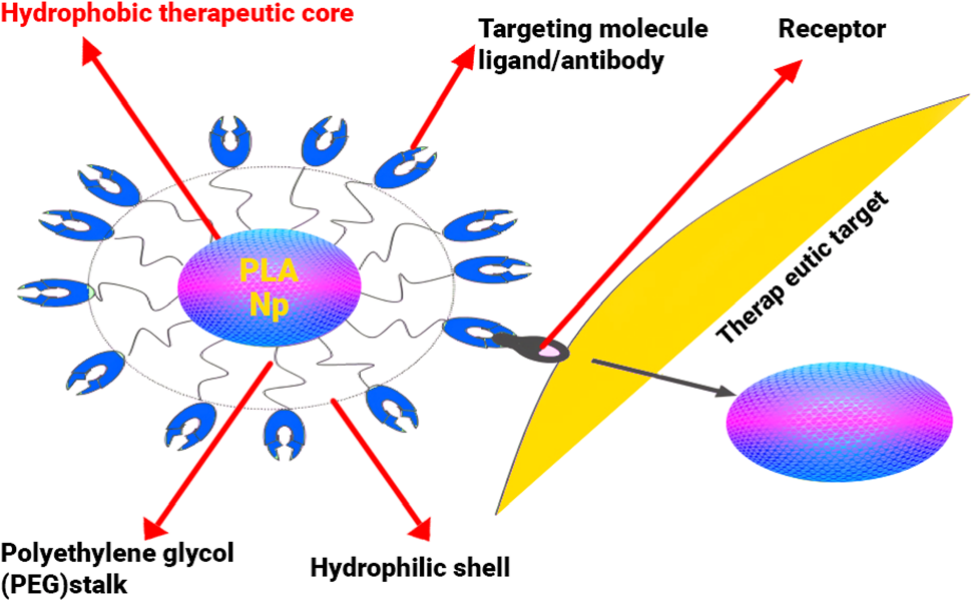
Schematic representation of a ligand-targeted polymeric nanoparticle based on polylactic acid (PLA). The hydrophobic core of the PLA nanoparticle encapsulates the therapeutic agent, while a polyethylene-glycol (PEG) corona confers aqueous stability. Targeting ligands or antibodies are conjugated to the distal ends of the PEG chains, enabling specific recognition of cell-surface receptors. Upon receptor binding, the nanoparticle can traverse physiological barriers (*e.g.*, the blood-brain barrier) and release the encapsulated drug at the desired site, leveraging the high affinity and selectivity of the attached targeting moiety (peptide, protein, antibody, *etc.*).

Furthermore, PNPs can be readily functionalized to improve targeting and overcome biological barriers. Surface modification with specific ligands (*e.g.*, antibodies, aptamers) enables active targeting to desired tissues or cell types, enhancing therapeutic specificity.^154^ This is especially important for reaching difficult-to-access sites like the central nervous system (CNS), which requires getting past the protective blood–brain barrier (BBB) to adjust the body's central clock.^155^ Strategies to cross the BBB include using targeting molecules, optimizing the nanoparticle's surface charge, adding a PEG coating, or even using magnets for guidance^156^ (Fig. 10). Nanoparticles can also build up passively in certain tissues, especially tumors, by taking advantage of their leaky blood vessels (the EPR effect).^157^ The potential for targeted CNS delivery is exemplified by applications involving neuropeptides like orexin, which plays a role in sleep–wake regulation.^158^

Advances in polymer chemistry and materials science underpin these capabilities. PLGA, an FDA-approved, biodegradable polyester, remains a cornerstone material due to its well-established safety profile and tunable degradation rates.^159^ Sophisticated polymerization techniques, such as reversible addition-fragmentation chain-transfer (RAFT) polymerization, afford precise control over polymer architecture, molecular weight, and composition.^132^ This molecular-level control translates into fine-tuning of nanoparticle characteristics like size, surface properties, and, importantly, the rate of drug release,^160^ which is essential for tailoring delivery to specific chronotherapeutic schedules. Illustrating this potential, when paclitaxel-loaded PNPs were given on a timed schedule (15 hours after sunrise), they were more effective against lung cancer cells than the free drug given at a random time.^161^ Computational studies further support chronotherapeutic approaches, indicating that circadian variations in hemodynamics (*e.g.*, lower shear forces during sleep) can enhance nanoparticle retention within the vasculature.^90^ Complex formulations, such as PEGylated PNPs co-delivering paclitaxel and survivin-targeting siRNA, also demonstrate the platform's capacity for combination therapy with reduced toxicity and enhanced efficacy.^162^ Collectively, the high degree of tunability makes PNPs powerful tools for developing advanced drug delivery strategies, including those required for effective chronotherapy.

## 5. Applications of nanomaterial-enabled circadian strategies

Having established how nanotechnology provides a powerful toolbox for timed and targeted drug delivery, we now shift our focus to what these platforms can achieve in medicine. The true test of these lipid, polymeric, and hybrid systems lies in their real-world application. This section explores how these advanced tools are being translated into specific strategies to directly modulate core clock genes, optimize cancer therapy, and manage complex disorders tied to circadian disruption, including metabolic syndrome, cardiovascular disease, and neurodegenerative conditions. These examples move beyond theory, demonstrating the tangible promise of merging circadian biology with nanomedicine to create more effective and personalized treatments.

### 5.1 Modulating the molecular clock *via* targeted nano-delivery

**Fig. 11:**
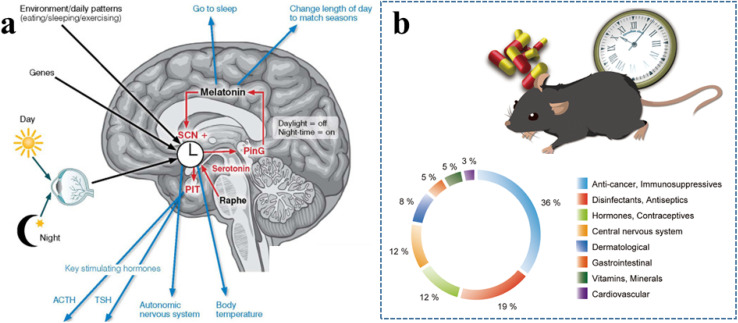
Polymeric nanoparticle (PNP) strategies for circadian-rhythm regulation and targeted drug delivery: (a) Schematic of the central circadian circuitry in the human brain. The suprachiasmatic nucleus (SCN) receives photic input from the retina and integrates genetic and environmental cues (*e.g.*, feeding, sleep, exercise). It orchestrates downstream pathways that control melatonin release, serotonergic signalling from the raphe nuclei, pituitary hormone secretion (ACTH, TSH), autonomic output, and body-temperature rhythms, thereby governing sleep–wake cycles and seasonal adjustment of day-length perception.^245^ (b) Overview of circadian-modulating drugs identified among approved pharmaceuticals. A circular diagram displays the therapeutic classes of 59 validated hit compounds that show dose-dependent effects on circadian parameters in secondary assays.^164^

Because the circadian clock is a master regulator of the body and its disruption is linked to many diseases, directly adjusting its core molecular machinery is a promising therapeutic strategy. Consequently, there is growing interest in pharmacological interventions targeting the clockwork itself.^163^ This pursuit primarily follows two avenues: the repurposing of existing drugs and the development of novel, targeted small-molecule modulators. Drug repositioning involves identifying approved drugs, originally developed for other conditions, that also happen to affect the circadian clock. This approach offers a faster path to the clinic because these drugs already have established safety and pharmacokinetic data. Indeed, various classes of existing drugs, including certain hormones, anticancer agents, and CNS drugs, have been identified as possessing clock-modulating properties^164^ (Fig. 11). In parallel, significant research efforts focus on discovering and designing novel small molecules specifically targeting core clock components (*e.g.*, *BMAL1*, *CLOCK*, *PER*, *CRY*) or their regulatory enzymes (*e.g.*, kinases like CK1δ/ε) with high specificity and potency.^165,166^ These development programs typically employ high-throughput screening, structure-based design, and detailed characterization in relevant circadian models.^167,168^

A great clinical example proving this approach can work is the use of lithium for bipolar disorder. Lithium, a long-established mood stabilizer, demonstrably lengthens the circadian period and alters clock gene expression.^169,170^ This evidence strongly suggests that lithium works, at least in part, by directly affecting the molecular clock. Beyond the delivery of conventional pharmacological agents, certain nanomaterials themselves are emerging as potential direct modulators of circadian function. Distinct from their role as carriers, specific types of nanoparticles (*e.g.*, metallic, iron oxide) have been shown to interact with and influence the activity of core clock proteins like *CLOCK* and *BMAL1*, or associated regulatory elements such as microRNAs.^43^ Furthermore, nanomaterials can be engineered to facilitate highly localized delivery of specific clock modulators (*e.g.*, inhibitors of casein kinase 1δ/ε) directly to target cells or tissues, offering precise spatial control over rhythm modulation.^171^ Additionally, nanomaterials present opportunities to optimize the delivery or perception of light, a primary environmental cue (zeitgeber) for the circadian system. This could involve materials enhancing light penetration or mimicking specific light spectra, potentially offering strategies to mitigate circadian disruption linked to inadequate light exposure, which is relevant for metabolic health and other areas.^172,173^ These approaches represent exciting new ways for nanotechnology to directly interact with the body's biological timing systems.

### 5.2 Nano-enhanced cancer chronotherapy

Cancer chronotherapy uses the principles of circadian biology to optimize cancer treatment schedules, representing a major strategic advance in oncology. This approach recognizes that fundamental biological processes including cell cycle progression, DNA damage repair, drug metabolism pathways, and immune surveillance exhibit robust circadian oscillations within both healthy tissues and neoplastic cells.^86,174^ By aligning the administration of anticancer agents with specific times of day, cancer chronotherapy aims to enhance the therapeutic index, maximizing drug efficacy against tumor cells while minimizing toxicity to normal host tissues.^175,176^

A growing body of clinical evidence confirms the benefits of this timed approach. For instance, the efficacy and toxicity profiles of widely used chemotherapeutics like irinotecan and 5-fluorouracil demonstrate significant circadian dependency.^177^ In metastatic colorectal cancer patients, administering these agents according to individualized chronomodulated schedules has been associated with improved treatment responses, reduced incidence and severity of adverse effects, and even enhanced overall survival compared to conventional, non-timed administration protocols.^178–181^ Furthermore, monitoring patient circadian rhythms during treatment has shown predictive value for clinical outcomes and toxicity susceptibility, reinforcing the clinical relevance of biological timing.

Critically, for cancer chronotherapy to be effective, it must be personalized. There is significant variation in daily rhythms from person to person (known as chronotypes), which means a single “best” time to give a drug is unlikely to work for everyone.^182,183^ Therefore, integrating patient-specific biomarkers ranging from core body temperature rhythms to peripheral clock gene expression or wearable sensor data is essential for tailoring treatment schedules to individual physiology and maximizing the benefits of chronotherapy.^184^ This shift towards personalized timing aligns with broader trends in precision oncology.

**Fig. 12:**
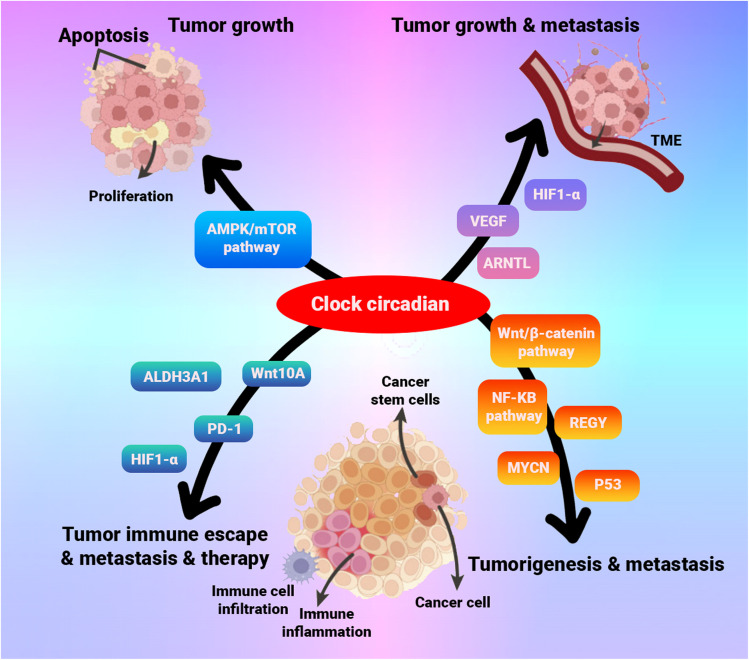
Schematic illustration of circadian-clock-driven mechanisms that contribute to cancer pathogenesis. The clock regulates tumor growth by balancing proliferation and apoptosis through the AMPK/mTOR signalling axis, while it promotes tumor progression and metastasis by stimulating angiogenesis (VEGF), metabolic reprogramming (HIF-1α), and remodelling of the tumour microenvironment, together with activation of pathways such as Wnt/β-catenin and ARNTL. In cancer stem cells, circadian cues control self-renewal and differentiation via networks that include MYCN and p53. Finally, the clock influences immune evasion and therapy resistance by modulating immune-cell infiltration (PD-1), detoxification enzymes (ALDH3A1), and inflammatory signalling (NF-κB), thereby facilitating escape from immune surveillance and reduced therapeutic efficacy.

The reason for timing cancer therapy goes beyond simply optimizing how the body processes a drug. Growing evidence indicates that disruption of the body's endogenous circadian system itself is linked to increased cancer incidence and poorer prognoses.^185^ This suggests that maintaining robust circadian rhythms may contribute to cancer prevention, and therapeutic strategies that reinforce or restore circadian synchrony could potentially inhibit tumor progression.^185^ Underlying these connections are intricate molecular interactions between the core circadian clock machinery and key cancer pathways. Core clock proteins, such as *BMAL1*, directly influence tumorigenesis and modulate cancer cell sensitivity to treatments like paclitaxel.^186^ Similarly, the *CLOCK* protein has been implicated in regulating immune cell interactions within the tumor microenvironment (*e.g.*, involving CD8+ T cells) and pathways associated with metastasis (*e.g.*, Wnt10A, ALDH3A1).^187^ A disrupted clock may also help cancer cells hide from the immune system, potentially involving immune checkpoints like PD-1 (ref. 188) (Fig. 12), highlighting the deep integration of circadian timing with cancer biology and immunology.

### 5.3 Nano-chronotherapeutic approaches for metabolic and cardiovascular disorders

**Fig. 13:**
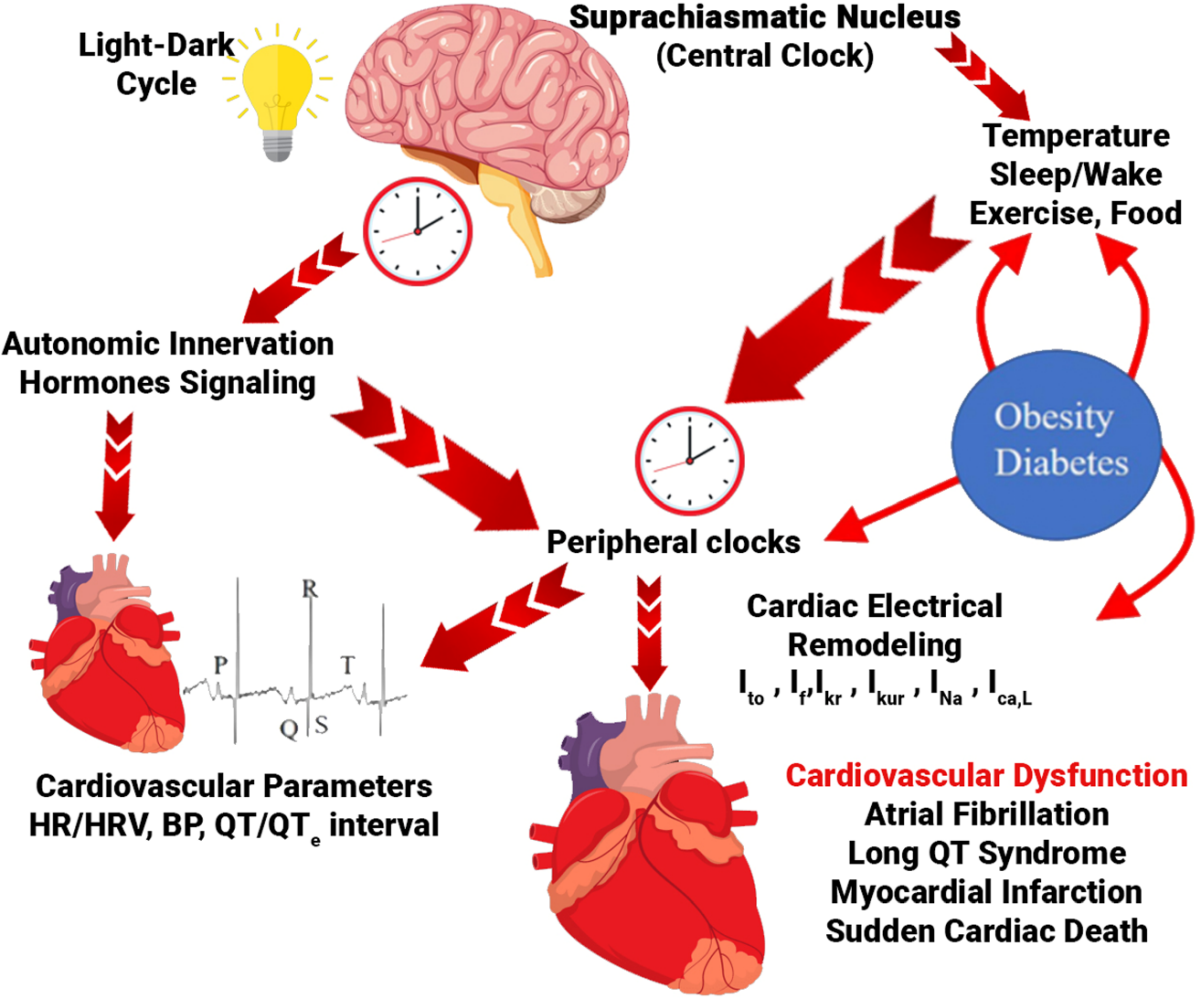
Diagram illustrating how circadian rhythms govern cardiovascular function. The light/dark cycle sets the central clock in the SCN, which subsequently drives rhythmic oscillations in peripheral tissue clocks *via* neurohumoral pathways. Peripheral clocks are also influenced by factors such as sleep/wake cycles, eating habits, physical activity, and temperature variations. In the heart, these processes lead to the rhythmic regulation of physiological functions (such as cardiovascular parameters and ion channel expression) as well as pathological mechanisms (like cardiovascular disease and arrhythmias). External factors, such as high-fat diets and shift work, may modify these influences. Additionally, metabolic conditions (*e.g.*, obesity and diabetes) can impact circadian rhythms across various tissues, particularly the heart, potentially leading to altered ion channel expression and a heightened risk of cardiovascular disease (CVD) and arrhythmias. Abbreviations: HR, heart rate; HRV, heart rate variability; BP, blood pressure; Ito, transient outward potassium current; If, funny current; IKr, rapid delayed rectifier potassium current; IKur, ultra-rapid delayed rectifier potassium current; INa, sodium current; ICaL, L-type calcium current.

A substantial body of evidence implicates circadian rhythm disruption as a critical factor in the pathophysiology of metabolic and cardiovascular diseases, which constitute a major global health burden. Changes in clock gene expression or a mismatch between our internal clock and the environment (*e.g.*, from shift work or irregular eating) can harm the body's ability to control blood sugar, alter how it processes fats, promote long-term inflammation, and damage the lining of blood vessels all of which contribute to heart and metabolic diseases. For example, disturbed circadian blood pressure patterns (*e.g.*, non-dipping hypertension) are associated with increased risk of target organ damage and adverse cardiovascular events.^189^ Furthermore, populations experiencing chronic circadian disruption, such as shift workers, exhibit significantly higher rates of myocardial infarction and stroke, likely driven by dysregulation of the autonomic nervous system and altered vascular function.^190–193^ Perturbations of circadian timing can also negatively impact cardiac repair mechanisms following ischemic injury.^194,195^ A schematic is shown in Fig. 13, which shows the circadian mechanism.^196^

Given this intimate relationship between biological timing and cardiometabolic health, strategies aimed at restoring or reinforcing circadian rhythms represent promising therapeutic avenues. Lifestyle interventions, such as time-restricted feeding (TRF), which aligns nutrient intake with the endogenous circadian clock, have demonstrated benefits for metabolic parameters and cardiovascular risk factors.^197,198^ Building upon these principles, pharmacological chronotherapy seeks to optimize medication timing based on known circadian variations in drug action or disease activity. This is already standard practice for some drugs, including cholesterol-lowering statins and various blood pressure medications, where the time of day they are taken significantly affects how well they work and how well they are tolerated.^199^ Additionally, research is actively pursuing novel small-molecule modulators designed to directly target core components of the molecular clock as potential treatments for circadian misalignment and related disorders.^165,192^

Nanotechnology offers powerful tools to refine and enable these chronotherapeutic approaches for metabolic and cardiovascular diseases. NDDSs are particularly advantageous for achieving the precise temporal control required for effective chronotherapy. Utilizing biocompatible and biodegradable polymers such as PLA, polyglycolic acid (PGA), and their copolymer PLGA, NDDSs can be engineered for controlled, sustained, or triggered release profiles, matching drug availability to optimal therapeutic windows.^105,106^ This capability is crucial for delivering agents like insulin or statins in alignment with circadian metabolic fluctuations. Moreover, nanotechnology provides innovative solutions for challenging conditions like peripheral arterial disease (PAD), offering platforms for targeted drug delivery, enhanced imaging capabilities, and systems that can both diagnose and treat a condition (nanotheranostics).^105^ All of these could potentially be combined with chronotherapeutic timing principles to maximize patient outcomes.

### 5.4 Addressing sleep disorders with timed and targeted nano-delivery

Sleep disorders, encompassing conditions like insomnia and narcolepsy, represent a significant public health issue frequently associated with disruptions in the endogenous circadian timing system. The SCN and the hormones it controls, especially melatonin, are essential for directing the normal sleep–wake cycle. Therefore, problems with this system caused by genetics, environmental factors, or other health conditions can lead to a wide range of sleep problems.^200^ While traditional hypnotic medications are widely used, they often provide only symptomatic relief, may not correct underlying circadian misalignment, and can be associated with adverse effects such as residual daytime sedation or cognitive impairment.^201^

Recognizing these limitations, chronotherapeutic strategies offer a more physiologically attuned approach. By timing interventions to align with the body's natural rhythms, chronotherapy aims to improve efficacy and tolerability.^202^ This principle extends beyond simple timing adjustments to include therapies directly targeting core sleep–wake regulators. Melatonin administration, for instance, is used to reset or reinforce circadian phase, proving beneficial in certain sleep disorders, particularly those comorbid with neurodegenerative conditions like Parkinson's disease, where it may also exert neuroprotective effects.^203,204^ Conversely, modulating the orexin system, a key promoter of wakefulness, offers another targeted strategy. Drugs that block orexin receptors are used to help people sleep, while drugs that activate them are being studied to treat disorders of excessive daytime sleepiness like narcolepsy.^205^

**Fig. 14:**
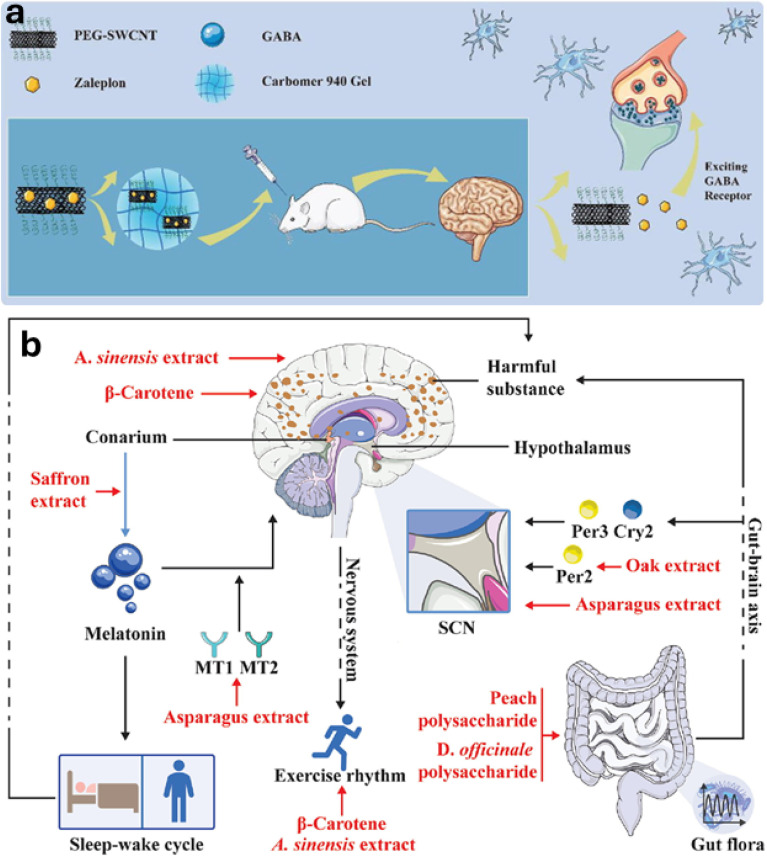
Nanotechnology- and natural-product-based strategies for circadian modulation of sleep and neuroprotection. (a) Schematic of a polyethylene-glycol-functionalised single-walled carbon nanotube (PEG-SWCNT) delivery platform for insomnia therapy. Zaleplon, a GABA A-receptor agonist, and γ-aminobutyric acid (GABA) are co-encapsulated within a Carbomer 940 hydrogel matrix that is loaded onto the PEG-SWCNT carrier. The nanocomposite enables targeted delivery to the central nervous system, augments GABAergic neurotransmission and thereby normalises the sleep–wake cycle.^207^ (b) Conceptual diagram of how selected natural products exert neuroprotective effects by modulating circadian biology. Representative compounds—including β-carotene, Angelica sinensis extract, saffron extract, asparagus extract and peach polysaccharide—act through multiple, inter-related mechanisms: (i) direct regulation of core clock genes (*e.g.*, PER2, CRY2) within the suprachiasmatic nucleus; (ii) modulation of melatonin synthesis and signalling *via* MT1/MT2 receptors; (iii) influence on gut-brain axis communication; and (iv) optimisation of behavioural rhythms such as sleep–wake cycles and physical activity. Collectively, these actions mitigate the accumulation of neurotoxic substances and prevent pathological remodelling of neural circuits.^210^

Nanotechnology provides powerful tools to enhance the delivery and effectiveness of these chronotherapeutic and targeted approaches for sleep disorders. Advanced drug delivery systems can overcome the pharmacokinetic limitations of sleep medications. For example, self-nano-emulsifying drug delivery systems (SNEDDS) have been developed to improve the solubility and oral bioavailability of poorly absorbed hypnotics like zaleplon,^206^ potentially leading to more consistent therapeutic effects and improved patient compliance. Furthermore, nanotechnology enables novel administration routes and release profiles. For instance, one study used a nasal spray with modified carbon nanotubes to deliver the drug zaleplon. This method achieved rapid brain entry to help with falling asleep, followed by a slower release to improve staying asleep. The system also included a special gel designed to minimize grogginess the next day^207^ (Fig. 14a). Transdermal systems incorporating digital automation also offer potential for precise spatiotemporal control over drug delivery, which could be advantageous for managing chronic sleep conditions.^208^

More sophisticated strategies involve feedback-controlled systems. Research is exploring adaptive platforms that adjust drug release based on real-time body signals, such as brain activity measured by an EEG.^209^ Such systems could personalize therapy, optimizing sleep quality by delivering medication only when needed while preserving natural sleep architecture during periods of adequate endogenous sleep drive. Underpinning the rationale for these advanced interventions is the growing understanding of the molecular links between the circadian clock and sleep regulation. Impaired expression of core clock genes like *BMAL1* is associated with inflammation, oxidative stress, and vascular dysfunction, factors contributing not only to sleep disturbances but also to related comorbidities like atherosclerosis.^210^ (Fig. 14b). Therefore, innovative delivery systems capable of targeting these fundamental circadian regulatory mechanisms represent a promising frontier for developing more effective and personalized treatments for sleep disorders.^211^

### 5.5 Addressing mental health and neurodegenerative diseases *via* nano-enabled chronotherapeutics

**Fig. 15:**
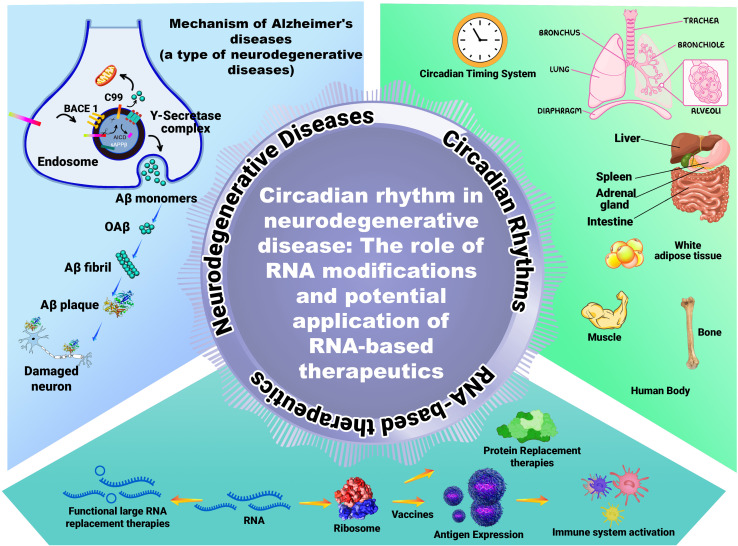
Highlights the role of RNA modifications in circadian rhythm-regulated neurodegenerative diseases and presents the potential applications.

There is a strong two-way relationship between the health of the circadian clock and the health of the brain, affecting both mental well-being and neurodegenerative diseases. Disruptions to the circadian system, whether stemming from lifestyle, environmental factors, or pathology, are strongly correlated with increased susceptibility to mood disorders like depression and anxiety.^186,212^ Conversely, interventions aimed at stabilizing circadian rhythms can alleviate symptoms of these conditions.^213,214^ Key neurobiological systems implicated in this link include the melatonin signaling pathway,^215^ crucial for sleep–wake regulation, and the serotonergic system,^216^ itself under circadian influence and vital for mood regulation. Similarly, compelling evidence connects circadian dysfunction with the pathogenesis and progression of major neurodegenerative diseases, including AD, PD, and Huntington's diseases.^217^ These circadian disturbances are more than just symptoms; they are now seen as contributing factors that can worsen brain inflammation, oxidative stress, and other harmful molecular changes.^218,219^ Neurodegenerative diseases are progressive impairments of the central or peripheral nervous system, often linked to genetic and biochemical factors. These include synaptic and neuronal deficits, abnormal protein homeostasis, DNA and RNA defects, inflammation, and pathological protein aggregation. Circadian rhythms, such as those in Alzheimer's, Parkinson's, and Huntington's diseases, are affected by oxidative stress, neuroinflammation, and other mechanisms. Disruptions in circadian rhythms, often linked to alterations in RNA modifications, contribute to disease progression. Current research on neurodegenerative diseases focuses on the relationship between aberrant circadian rhythm, RNA modifications, and potential applications of RNA-based drugs, as illustrated in Fig. 15.

Given these intricate links, targeting the circadian system presents a compelling therapeutic strategy for both psychiatric and neurodegenerative disorders. However, a principal challenge in treating these conditions lies in effectively delivering therapeutic agents across the BBB. Nanotechnology-based drug delivery systems offer promising strategies to overcome this obstacle. Engineered platforms such as SLNs, NLCs, and PNPs can be designed to facilitate BBB transport, enhance drug bioavailability within the CNS, and enable targeted delivery to specific neural cell populations or brain regions.^220,221^ This improved delivery is beneficial not only for conventional pharmaceuticals but also for potentially therapeutic natural compounds derived from medicinal plants, which often suffer from poor CNS penetration and bioavailability; nano-formulations can mitigate these limitations.^222^ Furthermore, the versatility of platforms like PNPs allows for the potential co-delivery of multiple agents to address the complex, multifaceted nature of many neurodegenerative conditions.^223^

The principles of chronopharmacology, as discussed in earlier sections, are highly relevant in this context. Drug delivery systems designed to synchronize therapeutic effects with circadian rhythms (*e.g.*, modified-release formulations) can enhance treatment efficacy and patient compliance, particularly for chronic conditions requiring consistent therapeutic drug levels.^224^ Therefore, targeting the circadian system, particularly through the use of sophisticated drug delivery systems, holds significant promise for improving the lives of individuals affected by these challenging conditions.

## 6. Challenges and limitations

While the strategies discussed above show great promise, moving them from the lab to the clinic presents critical hurdles. The complexity of human circadian biology, coupled with interindividual variability and fluctuating physiological states, poses significant challenges to achieving consistent therapeutic efficacy. Furthermore, as these strategies increasingly target specific biological windows for optimized treatment outcomes, factors such as biological barriers, timing accuracy, and systemic bio-distribution become central concerns. This section analyzes these limitations, including how the body's daily rhythms can affect a drug's performance, why personalized timing is essential, and the evolving regulatory rules for approving these advanced nanomedicines. Addressing these issues is essential for refining current technologies and ensuring their successful integration into mainstream clinical practice.

### 6.1 Biological barriers and the influence of circadian rhythms on delivery system efficacy

A treatment's success largely depends on whether the drug delivery system can get past the body's natural biological barriers, which work to maintain balance and protect tissues. Barriers such as the BBB, the GI tract lining, and the skin significantly impede drug transport, limiting bioavailability and therapeutic efficacy. Critically, the function and permeability of these barriers are not constant; they exhibit dynamic fluctuations governed by the endogenous circadian timing system. As a result, how well a drug delivery system works can depend heavily on the time of day it is given.^225^

The BBB, for instance, is a highly selective interface protecting the CNS, and its permeability undergoes circadian oscillations.^226^ This rhythmic variation directly impacts the ability of therapeutic agents, including those delivered *via* nanoparticles, to access the brain parenchyma. Ignoring these fluctuations can lead to suboptimal drug exposure within the CNS at certain times, potentially compromising the treatment of neurological and psychiatric disorders. Similarly, the physiology of the GI tract, the primary site for oral drug absorption, is under pronounced circadian control.^227^ Daily rhythms in stomach emptying, gut movement, digestive enzyme activity, and drug-transporting proteins all influence how quickly and completely a drug is absorbed. This creates daily variations in the amount of available drug for orally administered medicines.^17^

This same principle of daily rhythm applies to other delivery routes. Transdermal delivery faces the skin barrier, whose permeability also fluctuates daily, thereby affecting the absorption rate of topically applied therapeutics.^228^ Beyond these physical barriers, systemic pharmacokinetic processes are deeply intertwined with circadian rhythms. Drug metabolism and clearance are significantly influenced by the rhythmic activity of hepatic and extrahepatic drug-metabolizing enzymes, often regulated by circadian hormonal signals like glucocorticoids.^229^ Disruption of these metabolic rhythms is linked to various pathologies,^230,231^ and the time-dependent variation in enzyme activity can alter drug half-life and exposure. Furthermore, the immune system exhibits robust circadian oscillations in cell trafficking, activation, and inflammatory responses, which can impact the efficacy and toxicity of immunotherapies and treatments for inflammatory conditions.^232^

These pervasive circadian influences on biological barriers and physiological processes present both a significant challenge and a compelling opportunity for drug delivery. The challenge lies in accounting for this inherent temporal variability; failure to do so can result in unpredictable PK, reduced therapeutic efficacy, and potentially increased risk of adverse effects. The opportunity, however, arises from harnessing this knowledge through chronopharmacology. By strategically timing drug administration or employing sophisticated delivery systems, such as pulsatile drug delivery systems (PDDS),^233^ designed to release drugs in synchrony with specific circadian phases, it is possible to optimize therapeutic outcomes, enhancing efficacy while minimizing toxicity, particularly within the context of chronotherapy.^174,233^

### 6.2 Variability in circadian rhythms across individuals

A major challenge for using chronotherapy widely is that daily rhythms vary significantly from person to person. While everyone has an internal clock that runs on a roughly 24-hour cycle, key features of these rhythms, like their exact timing, strength, and consistency, differ considerably among individuals.^6^ This inter-individual variability arises from a complex interplay of genetic, epigenetic, environmental, and lifestyle factors, complicating the development of universally effective circadian-based therapeutic strategies.

Our genes play a significant role in this variability, though we are still learning the full extent of their influence. Polymorphisms or differential expression of core clock genes and their regulators can alter the circadian period or phase. For instance, transcription factors like KLF7 influence clock gene expression within the SCN, impacting circadian behavior.^234^ Post-transcriptional regulation, involving elements such as microRNAs (*e.g.*, miR-122), adds another layer of complexity and potential individual variation.^235,236^ Furthermore, novel regulatory interactions continue to be uncovered; for example, the protein PIWIL2 has been shown to modulate the stability and activity of core clock components *BMAL1* and *CLOCK*, representing another mechanism that could contribute to individual differences in circadian function.^237^

Beyond genetic predisposition, environmental cues (zeitgebers) and behavioral choices exert profound effects on circadian timing. Light exposure, particularly its timing, intensity, and spectral composition, acts as the primary synchronizing signal for the master clock in the SCN, mediated *via* intrinsically photosensitive retinal ganglion cells (ipRGCs) expressing melanopsin.^238^ Individual differences in daily light exposure patterns are therefore a major source of variation in circadian phase. Feeding schedules represent another powerful zeitgeber, especially for peripheral clocks in metabolic tissues like the liver.^239^ The timing of meals relative to the internal clock significantly impacts metabolic rhythms, and aligning both light exposure and feeding patterns is crucial for robust circadian entrainment and metabolic health.^240^

Consequently, the pronounced variability in circadian rhythms across the population presents a fundamental challenge for implementing effective chronotherapeutic strategies. A standardized, “one-size-fits-all” timing for drug administration or circadian modulation is unlikely to yield optimal results for all individuals. This necessitates a shift towards personalized chronotherapy, integrating assessments of individual circadian characteristics (chronotype and phase) derived from robust biomarkers or wearable sensor data. Furthermore, it highlights the need for adaptable drug delivery systems that can be tailored to meet the specific timing needs of each patient, thereby maximizing the potential benefits of circadian-informed treatments.

### 6.3 Regulatory considerations and safety profiles of circadian interventions

While therapeutically promising, interventions targeting the circadian system necessitate careful consideration of potential safety issues and regulatory challenges, owing to the system's complexity and pervasive physiological influence. Concerns encompass both the risks inherent in directly modulating the core clock machinery and the practical challenges associated with implementing time-based therapies like chronotherapy. A primary safety consideration stems from the intricate and pleiotropic nature of the molecular clockwork. Direct pharmacological manipulation of core clock components, while aiming for therapeutic benefit, carries an inherent risk of unintended consequences due to the disruption of numerous interconnected, clock-controlled pathways essential for homeostasis.^241^ Given the profound influence of the circadian system on drug metabolism, disposition, and overall metabolic health,^242^ treatments that directly target the clock must be carefully tested for potential off-target effects that could be unsafe or interfere with other medications.

Chronotherapy, the strategy of optimizing drug administration timing relative to circadian rhythms, also presents specific safety considerations despite its potential to enhance the therapeutic index, particularly in oncology. A critical challenge is the potential for misalignment between a standardized or empirically derived dosing schedule and an individual's unique circadian phase. Such misalignment could theoretically negate the benefits of timing, potentially reducing treatment efficacy or even increasing toxicity compared to non-timed administration.^243^ Mitigating these risks requires robust strategies incorporated early in development and clinical implementation. Addressing the significant inter-individual variability in circadian timing (as discussed in Section 6.2) is paramount. This necessitates the development and validation of reliable, accessible biomarkers capable of accurately assessing individual circadian phase to enable personalized chronotherapeutic schedules. Furthermore, careful dose optimization within timed regimens and thorough evaluation *via* well-designed, long-term clinical studies are essential to establish the safety and efficacy profiles of circadian-based interventions, especially for chronic disease management.^242^ Integrating these personalized approaches will be crucial for responsibly translating the potential of circadian medicine into safe and effective clinical practice.

## 7. Future prospects

The convergence of circadian biology research, advanced drug delivery technologies, and the principles of precision medicine heralds a transformative era in healthcare. As fundamental understanding of circadian rhythm influence on health and disease expands, coupled with technological advancements for monitoring and modulating these rhythms, the prospect of truly personalized chronotherapy, tailoring interventions to individual circadian profiles, becomes increasingly tangible. Realizing this vision necessitates overcoming key challenges, primarily the significant inter-individual variability in circadian timing, amplitude, and phase, which arises from diverse genetic, environmental, and lifestyle factors.^244^ This inherent variability underscores the limitations of a “one-size-fits-all” approach and mandates the development of personalized strategies. Central to this effort is the establishment of robust biomarkers and accessible monitoring technologies. Accurate, real-time assessment of an individual's circadian status, potentially through wearable sensors tracking physiological parameters (activity, temperature, *etc.*) or *via* validated molecular biomarkers in accessible biofluids, is essential for tailoring therapeutic timing effectively.

Complementary to precise assessment is the need for sophisticated drug delivery systems capable of executing personalized chronotherapeutic regimens. As detailed previously, advanced platforms utilizing nanoparticles, stimuli-responsive materials, and optimized administration routes (*e.g.*, transdermal) are critical for achieving the required temporal control over drug release. Future systems must be adaptable, capable of delivering diverse therapeutic modalities (from small molecules to biologics) according to individualized, potentially complex, timed schedules to maximize efficacy and minimize toxicity.

Beyond specific technologies, a systems-level understanding is vital for advancing circadian medicine. Comprehending the intricate interactions between the central and peripheral circadian clocks and other major physiological networks, such as the immune and metabolic systems, requires integrative approaches. This involves consolidating multi-omics data with physiological and behavioral measurements to construct comprehensive models of individual circadian function and dysfunction. Advanced computational tools, including machine learning and artificial intelligence algorithms, will be instrumental in analyzing these complex datasets and translating systems-level insights into clinically actionable strategies.

Ultimately, translating these advancements into routine clinical practice depends on rigorous validation and effective implementation. Large-scale clinical trials are imperative to demonstrate the safety and efficacy of personalized chronotherapeutic interventions across diverse patient populations and disease contexts. Concurrently, overcoming logistical barriers, educating healthcare providers, and ensuring patient access to necessary monitoring and delivery technologies will be crucial for integrating circadian medicine successfully into healthcare systems. As progress continues across these fronts, the potential for circadian-based strategies to revolutionize disease prevention and treatment will undoubtedly expand, paving the way for more effective, individualized healthcare solutions.

## Conflicts of interest

The authors declare that they have no known competing financial interests or personal relationships that could have appeared to influence the work reported in this paper.

## Data Availability

No primary research results, software or code have been included and no new data were generated or analysed as part of this review.
