# Correction: Nanomaterial-enabled drug delivery systems for circadian medicine: bridging direct rhythm modulation and chronotherapy

**DOI:** 10.1039/d5ra90137e

**Published:** 2025-11-19

**Authors:** Muhammad Zeeshan, Juncheng Hu, Chuan-Xi Mao, Almas Danish, Ying Xiong, Muhammad Sultan Irshad, Van-Duong Dao, Zhihua Liu

**Affiliations:** a State Key Laboratory of Biocatalysis and Enzyme Engineering, School of Life Sciences, Hubei University Wuhan P.R. China Ying.Xiong@hubu.edu.cn Zhihua_Liu@hubu.edu.cn; b Ministry of Education Key Laboratory for the Green Preparation and Application of Functional Materials, School of New Energy and Electrical Engineering, Hubei University Wuhan 430062 P.R. China muhammadsultanirshad@hubu.edu.cn; c Faculty of Biotechnology, Chemistry, and Environmental Engineering, Phenikaa School of Engineering, Phenikaa University Hanoi 12116 Vietnam duong.daovan@phenikaa-uni.edu.vn

## Abstract

Correction for “Nanomaterial-enabled drug delivery systems for circadian medicine: bridging direct rhythm modulation and chronotherapy” by Muhammad Zeeshan *et al.*, *RSC Adv.*, 2025, **15**, 31981–32008, https://doi.org/10.1039/D5RA04137F.

The authors regret an omission of a reference in the original article. Fig. 1 and 2 in the original article were reproduced from an article published by C. T. Butler, A. M. Rodgers, A. M. Curtis *et al.*^1^ The authors apologise for this error. The correct captions for Fig. 1 and 2 are displayed herein.

**Fig. 1:**
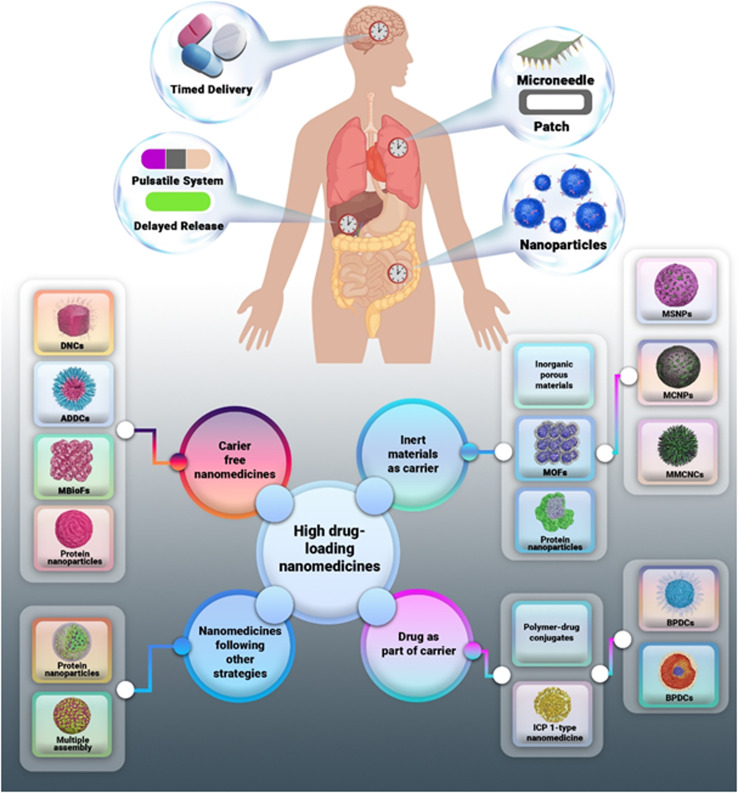
Schematic representation of tailored drug delivery strategies designed for targeted organ delivery and time-specific administration. The illustration depicts potential systems-such as timed release (brain), pulsatile or delayed systems (liver), microneedle patches (lungs), and nanoparticle-based delivery (intestine), as examples of emerging technologies that may, in the future, be aligned with circadian regulation of peripheral organs. This figure is conceptual and does not imply that organ-specific times for drug delivery systems are currently available or clinically established. It aims to highlight the growing potential of integrating circadian biology into advanced drug delivery design. Reproduced from C. T. Butler, A. M. Rodgers, A. M. Curtis *et al.*, Chrono-tailored drug delivery systems: recent advances and future directions, *Drug Deliv. and Transl. Res.*, 2024, **14**, 1756–1775 with permission from Springer Nature.

**Fig. 2:**
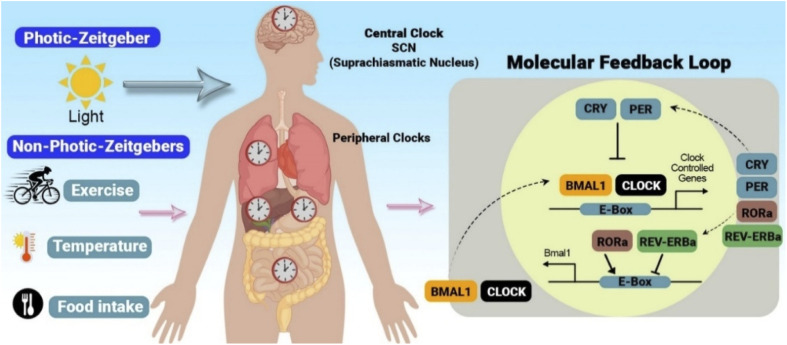
Integration of photic and non-photic zeitgebers by central and peripheral clocks. The SCN, the body's central circadian pacemaker, coordinates peripheral clocks throughout the body *via* neural and humoral signals. Photic zeitgebers primarily influence the SCN, while non-photic zeitgebers, such as exercise, sleep, temperature, and food intake, can act on both the SCN and peripheral clocks. This integrated input ensures the alignment of internal physiological processes with the external environment. At the molecular level, the core circadian clock mechanism involves a TTFL. In this loop, the *BMAL1* and *CLOCK* proteins heterodimerize and bind to E-box elements in the promoters of target genes, including *PER* and *CRY*, as well as other clock-controlled genes (CCGs), thereby inducing their transcription. *PER* and *CRY* proteins then accumulate, heterodimerize, and translocate back into the nucleus, where they inhibit *BMAL1-CLOCK* activity, thereby suppressing their transcription. Concurrently, *BMAL1* and *CLOCK* also regulate the expression of nuclear receptors retinoic acid receptor-related orphan receptor alpha (RORα) and REV-ERBα, which exert rhythmic activation and repression on *BMAL1* transcription, respectively, adding another layer of regulation to the core clock loop. The rhythmic expression of these core clock components and downstream CCGs drives circadian oscillations in a wide range of physiological processes, including metabolism, hormone secretion, and immune responses. Reproduced from C. T. Butler, A. M. Rodgers, A. M. Curtis *et al.*, Chrono-tailored drug delivery systems: recent advances and future directions, *Drug Deliv. and Transl. Res.*, 2024, **14**, 1756–1775 with permission from Springer Nature.

The Royal Society of Chemistry apologises for these errors and any consequent inconvenience to authors and readers.
